# Functional antibodies against *Plasmodium falciparum *sporozoites are associated with a longer time to qPCR-detected infection among schoolchildren in Burkina Faso

**DOI:** 10.12688/wellcomeopenres.14932.2

**Published:** 2019-05-01

**Authors:** Aissata Barry, Marije C. Behet, Issa Nébié, Kjerstin Lanke, Lynn Grignard, Alphonse Ouedraogo, Issiaka Soulama, Chris Drakeley, Robert Sauerwein, Judith M. Bolscher, Koen J. Dechering, Teun Bousema, Alfred B. Tiono, Bronner P. Gonçalves

**Affiliations:** 1 Centre National de Recherche et de Formation sur le Paludisme, Ouagadougou, Burkina Faso; 2 Radboud Institute for Health Sciences, Radboud University Medical Center, Nijmegen, The Netherlands; 3 Department of Immunology and Infection, London School of Hygiene and Tropical Medicine, London, UK; 4 TropIQ Health Sciences, Nijmegen, The Netherlands

**Keywords:** malaria, sporozoites, antibodies, immunity, sterilizing, pre-erythrocytic, liver-stage

## Abstract

**Background: **Individuals living in malaria-endemic regions develop immunity against severe malaria, but it is unclear whether immunity against pre-erythrocytic stages that blocks initiation of blood-stage infection after parasite inoculation develops following continuous natural exposure.

**Methods: **We cleared schoolchildren living in an area (health district of Saponé, Burkina Faso) with highly endemic seasonal malaria of possible sub-patent infections and examined them weekly for incident infections by nested PCR. Plasma samples collected at enrolment were used to quantify antibodies to the pre-eryhrocytic-stage antigens circumsporozoite protein (CSP) and Liver stage antigen 1 (LSA-1). *In vitro* sporozoite gliding inhibition and hepatocyte invasion inhibition by naturally acquired antibodies were assessed using *Plasmodium falciparum *NF54 sporozoites. Associations between antibody responses, functional pre-erythrocytic immunity phenotypes and time to infection detected by *18S* quantitative PCR were studied.

**Results: **A total of 51 children were monitored. Anti-CSP antibody titres showed a positive association with sporozoite gliding motility inhibition (P<0.0001, Spearman’s ρ=0.76). *In vitro *hepatocyte invasion was inhibited by naturally acquired antibodies (median inhibition, 19.4% [IQR 15.2-40.9%]), and there were positive correlations between invasion inhibition and gliding inhibition (P=0.005, Spearman’s ρ=0.67) and between invasion inhibition and CSP-specific antibodies (P=0.002, Spearman’s ρ=0.76). Survival analysis indicated longer time to infection in individuals displaying higher-than-median sporozoite gliding inhibition activity (P=0.01), although this association became non-significant after adjustment for blood-stage immunity (P = 0.06).

**Conclusions: **In summary, functional antibodies against the pre-erythrocytic stages of malaria infection are acquired in children who are repeatedly exposed to *Plasmodium *parasites. This immune response does not prevent them from becoming infected during a malaria transmission season, but might delay the appearance of blood stage parasitaemia. Our approach could not fully separate the effects of pre-erythrocytic-specific and blood-stage-specific antibody-mediated immune responses *in vivo*; epidemiological studies powered and designed to address this important question should become a research priority.

## Introduction

The most advanced malaria vaccine, RTS,S (trade name, Mosquirix), induces immune responses that target *P. falciparum* circumsporozoite protein (CSP), and thereby the pre-erythrocytic stages of malaria, and has been shown to be partially effective in delaying the time to clinical malaria episodes ^1^. Alongside the RTS,S subunit vaccine, several other pre-erythrocytic stage vaccines are under development, based on subunit or whole-parasite vaccination ^2– 5^. Vaccination with the attenuated sporozoite vaccine PfSPZ resulted in a protective efficacy of ~48%, as quantified by differences in time to first positive blood smears in malaria-experienced adults in Mali ^6^. The results of this and other vaccine trials and the efficient immunisation of malaria-naive individuals with multiple infected mosquito bites while receiving chloroquine ^7^ contrast with the limited epidemiological evidence of naturally acquired functional immunity to *Plasmodium* pre-erythrocytic stages, which could be possibly linked to the lower number of sporozoites in natural parasite inoculations or to the frequency of host-vector contacts. Individuals living in malaria-endemic regions can develop naturally acquired immunity against severe malaria disease and death ^8, 9^, but it is unclear whether immunity that reduces, entirely or partially, the probability of blood-stage infection after parasite inoculation develops following natural exposure ^9^. The high incidence of blood-stage re-infection after effective anti-malarial treatment in adults living in malaria-endemic regions suggests that sterilizing immunity does not develop even after years of repeated infection ^10, 11^. Similarly, cohort studies that have analysed the relationship between age and risk of *P. falciparum* infection showed no evidence for complete protection against infection and conflicting evidence on whether naturally acquired immunity can result in a different time to patency ^12, 13^. One of the most detailed studies on this topic reported clear negative associations between age and the risk of clinical malaria or microscopy-detected malaria infection, but similar times to PCR-detected infection for all age groups. The study concluded no or very limited evidence for an age-dependent acquisition of immunity that protects from infection ^13^.

Given the interest in pre-erythrocytic vaccines, studies are needed to understand natural protective immune responses that target sporozoite and liver-stages of malaria infection. Here, we determine the associations between responses affecting sporozoite gliding motility, hepatocyte invasion and malaria infection risk assessed by weekly quantitative PCR (qPCR) in a cohort of schoolchildren from Burkina Faso exposed to intense malaria transmission.

## Results

### Study population and follow-up

Of the 58 school-aged children who were recruited and received treatment at enrolment, 6 were PCR-positive 3 weeks after dihydroartemisinin-piperaquine (DHA-PQ) administration and were not eligible to continue follow-up. One child who was only followed for one routine visit, when no infection was detected, and who withdrew from the study, did not have immune responses quantified and was not included in this analysis.

**Figure 1.**
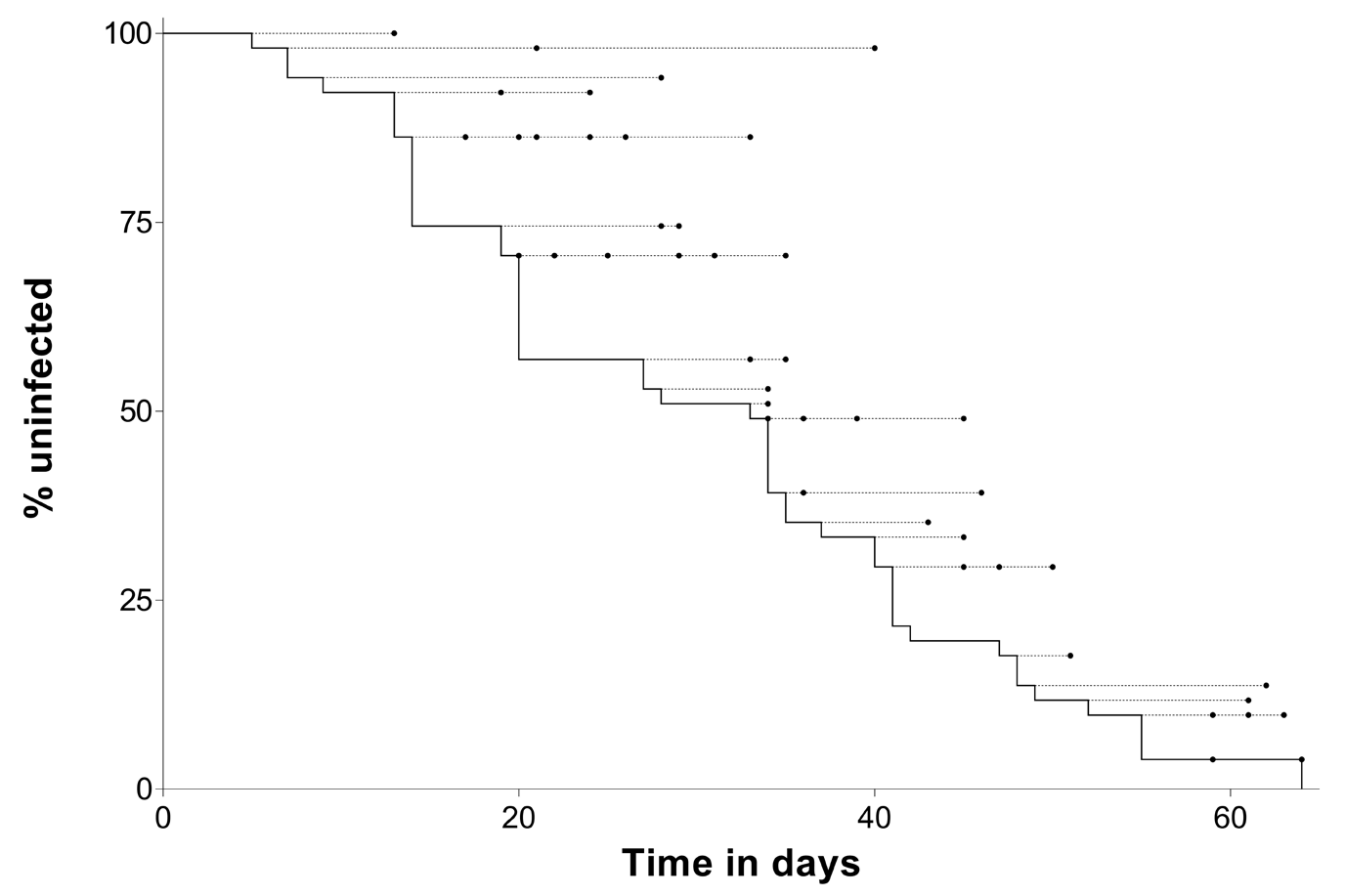
Time to malaria infection detection. Time-to-infection data from all study participants (N=51) are shown. The black line represents the percentage of the study population that remained uninfected at different time-points (y-axis). Circles indicate when individuals with first parasite detection at the start of the corresponding dashed line developed clinical symptoms. Time, x-axis, is relative to the confirmation of parasite clearance, 3 weeks after anti-malarial administration.

Parasitological and immunological data from the remaining 51 children followed intensively were analysed ( Table 1). Every week these study participants were screened for incident infections. All but one study participant had *P. falciparum* parasites detected by *18S* qPCR during follow-up. Malaria infection caused clinical disease in 43/50 children. One child developed symptomatology suggestive of malaria, but no parasites were detected in samples collected before and during the clinical episode; data from this child were censored after the onset of symptoms. The median time from confirmation of the absence of parasites (i.e. 3 weeks after anti-malarial treatment) to infection detection by nested PCR or onset of symptoms was 28 days; one child who did not have parasites detected by nested PCR was not included in this calculation. Similarly, the median time to parasite detection by *18S* qPCR was 30 days. In Figure 1, both the times of first *18S* qPCR positive result and, if applicable, of development of clinical disease are presented for all study participants.

**Table 1.** Study population.

Variable	Value
**Number of individuals**	
Screened and parasite-free by microscopy	58
Presence of parasites post-treatment	6
Consent withdrawn	1
Monitored	51
**Age in years, median (IQR)**	7.1 (5.7–8.1)
**Gender**	
Female, % (N)	37.2 (N=19)
**Reported bed net use, % (N)**	51.0 (26)
**Haemoglobin levels at the beginning of** follow-up *, median (IQR)	11.9 (11.3–12.5)
**Haemoglobin types**	
AA	70.6 (36)
AC	21.6 (11)
AS	5.9 (3)
SS	2.0 (1)
**Total number of weekly surveillance** **visits**	222
**Weekly visits/participant, median (IQR)**	4 (2–6)

*First weekly visit. IQR, interquartile range.

### Naturally acquired IgG and IgM antibodies targeting sporozoites

**Figure 2.**
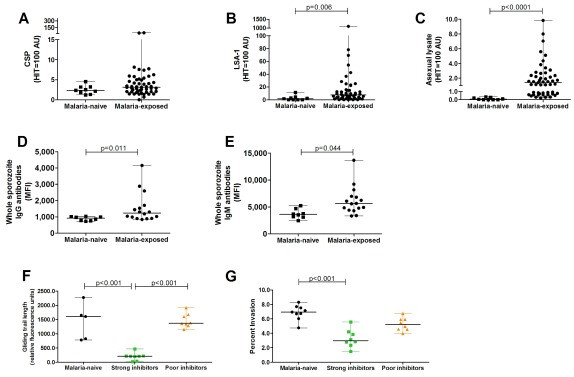
Naturally acquired pre-erythrocytic antibody levels and their functionality against sporozoite infectivity *in vitro*. Malaria antigen-specific antibody levels in children from Burkina Faso (n=51) and European malaria-naive adults (n=9) to the pre-erythrocytic antigens ( **A**) circumsporozoite protein (CSP), ( **B**) liver-stage antigen-1 (LSA-1), and ( **C**) asexual lysate as an internal control were determined by ELISAs and expressed as arbitrary units (AU). The amount of ( **D**) IgG and ( **E**) IgM antibodies recognizing sporozoites was determined by flow cytometry and shown as the geometric mean fluorescence intensity (MFI). To this end, *P. falciparum* NF54 sporozoites were pre-treated with 10% heat-inactivated plasma from children from Burkina Faso (N=16) and malaria-naive adults (N=8) and stained with fluorescently labelled antibodies against IgG and IgM antibodies. ( **F**) The gliding motility of *P. falciparum* NF54 sporozoites, pre-treated with plasma from children from Burkina Faso (N=51) and malaria-naive adults (N=5), was determined by *in vitro* gliding motility assays. Gliding trail length of sporozoites incubated with plasma from malaria-naive donors (N=5) or naturally exposed children who were poor (N=8) or strong (N=8) inhibitors of gliding motility are shown. ( **G**) The percent hepatocytes invaded by *P. falciparum* NF54 sporozoites pre-treated with plasma from children from Burkina Faso (N=16) who were shown to be either poor (n=8) or strong (n=8) gliding inhibitors and malaria-naive adults (N=9) was determined by *in vitro* invasion assays in human hepatoma cells. Comparisons between multiple groups were tested with Kruskal-Wallis test with Dunn’s multiple comparison post hoc test.

Malaria antigen-specific antibodies to pre-erythrocytic antigens CSP, liver stage antigen (LSA-1) and to asexual lysate were determined in naturally exposed children and malaria-naive European donors by ELISAs. Antibody titres to the CSP pre-erythrocytic antigen were on average low in naturally exposed children and not significantly different from malaria-naive donors ( Figure 2A, P=0.11; non-parametric tests were used for all comparisons), while LSA-1 antibody levels were significantly higher in malaria-exposed children compared to malaria-naive donors ( Figure 2B, P=0.006). As expected, asexual blood stage-specific antibody titers in naturally exposed children were higher compared to malaria-naive donors ( Figure 2C, P<0.0001). In addition to antigen-specific assays, IgG and IgM antibodies recognizing *Plasmodium* sporozoites were quantified using fluorescently labelled anti-IgG and anti-IgM antibodies by flow cytometric analysis. Levels of antibodies targeting whole sporozoites were significantly higher in the study participants compared to malaria-naive donors ( Figure 2D, E, P=0.01 and 0.04 for IgG and IgM, respectively). Strong correlations between IgG and IgM antibodies targeting whole sporozoites ( Figure S1A in Extended data ^14^, P=0.0005, Spearman’s ρ=0.75), and between CSP-IgG antibody levels and whole sporozoite IgG antibodies ( Figure S1B in Extended data ^14^, Spearman’s ρ=0.83, P<0.0001) were also observed.

### Naturally acquired antibodies in children neutralize *in vitro* sporozoite gliding motility

The neutralizing activity of naturally acquired antibodies against sporozoite motility was determined in an *in vitro* assay. Sporozoites pre-treated with PBS showed an average gliding trail surface of 7,392 (95% confidence interval [CI] 4,151-9,388) pixels. To characterize the effect of human plasma on sporozoite gliding motility that is independent of naturally acquired immunity, sporozoites were incubated in the presence of plasma from malaria-naive individuals (n=5) and showed an average gliding trail surface of 1,427(95% CI 646.6-2,207) pixels, significantly greater than the gliding trail surface of sporozoites incubated with 30 μg/ml of a monoclonal anti-CSP antibody, our positive control, 141 (95% CI 71.9-225.4) pixels.

Plasma from the majority of cohort participants reduced *in vitro* sporozoite gliding motility with a median gliding inhibition of 59.6% (IQR 27.8-77.0%); incubation of sporozoites with plasma from three participants resulted in lower gliding trail surfaces than the positive control. We defined two groups: one with poor gliding inhibition (individuals whose plasma inhibited less than 20% of sporozoite gliding motility, N=8) and the other with strong gliding inhibition (more than 80% gliding inhibition, N=8). The first group had similar gliding trail surface (median, 1,353; IQR, 1,311.7-1,639.2) compared to malaria-naive donors (median 1605.7, IQR 824.3 - 1,652) ( Figure 2F), whilst strong gliding inhibitors had a median trail surface of 186.2 (IQR 9.4 - 208.6), which was significantly lower compared to malaria-naive donors ( Figure 2F, P<0.01). *In vitro* gliding inhibition did not correlate with LSA-1 IgG antibody levels (P=0.11, Spearman’s ρ=0.23), but correlated with CSP-specific IgG antibodies ( Figure S2A in Extended data ^14^, P<0.0001, Spearman’s ρ=0.76), whole sporozoite IgG ( Figure S2B in Extended data ^14^, P<0.0001, Spearman’s ρ=0.81) and IgM antibodies ( Figure S2C in Extended data ^14^, P=0.01, Spearman’s ρ=0.61). While levels of IgG and IgM antibodies targeting whole sporozoite did not significantly differ between poor gliding inhibitors and malaria-naive adults, whole sporozoite antibody levels of strong gliding inhibitors were significantly higher compared to poor gliding inhibitors ( Figure S2D in Extended data ^14^, P<0.0001 and P=0.005 for IgG and IgM antibodies, respectively) and malaria-naive adults ( Figure S2D, E in Extended data ^14^, P<0.0001 and P=0.0006 for IgG and IgM antibodies, respectively). This suggests that quantitative differences in measured immune responses might explain variation in these functional phenotypes.

### Antibodies in malaria-exposed children neutralize *in vitro* sporozoite infectivity of hepatocytes

The inhibitory effect of naturally acquired antibodies on *in vitro* sporozoite invasion of hepatocytes was also assessed in a selected number of samples shown to inhibit gliding motility strongly (n=8) or poorly (n=8). *In vitro* invasion was inhibited by naturally acquired antibodies (median invasion inhibition, 19.4% [IQR, 15.2-40.9%]), and plasma from children categorized as strong gliding inhibitors (see previous section) also prevented hepatocyte invasion more effectively compared to malaria-naive donors ( Figure 2G, P<0.001). 7/8 plasma samples from strong gliding inhibitors reduced hepatocyte invasion by 20% or more, whereas only 2/8 poor gliding inhibitors inhibited at least 20% of hepatocyte invasion. There was a positive correlation between gliding and invasion inhibition ( Figure S3A in Extended data ^14^, P=0.005, Spearman’s ρ=0.67), suggesting that *in vitro* gliding inhibition by naturally acquired antibodies might serve as a good surrogate for *in vitro* hepatocyte invasion inhibition. Sporozoite invasion inhibition correlated with IgG targeting whole sporozoites ( Figure S3B in Extended data ^14^, P=0.004, Spearman’s ρ=0.67), but not with whole sporozoite IgM antibody levels ( Figure S3C in Extended data ^14^, P=0.13, Spearman’s ρ=0.38). There was a correlation of hepatocyte invasion inhibition with CSP-specific IgG antibodies ( Figure S3D in Extended data ^14^, P=0.002, Spearman’s ρ=0.76), but not with LSA-1-specific IgG antibodies (P=0.08, Spearman’s ρ=0.48).

### Evidence of natural risk-modifying pre-erythrocytic immunity

**Figure 3.**
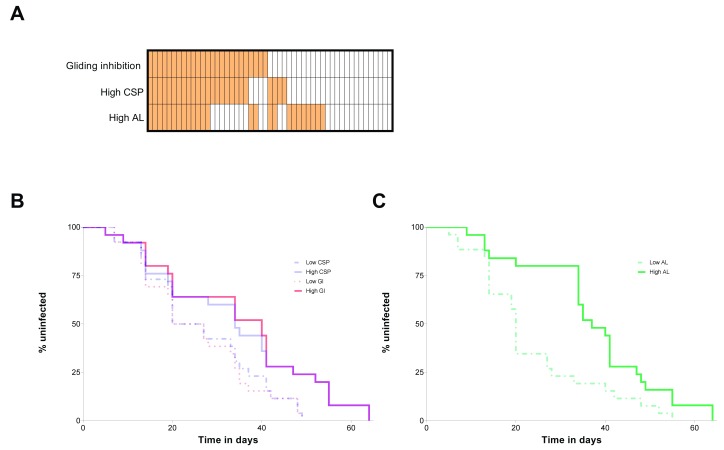
Effects of immune responses against liver- and blood-stage antigens on malaria infection risk. To assess the effect of immune responses on infection risk, children were classified based on whether the results of their assays were higher or lower than the study population median (see Results): in ( **A**), vertically aligned cells represent the same participant, and orange cells indicate that assay results are higher than the median (see also Figure S4, that shows a scatter plot of circumsporozoite protein (CSP) antibody levels and immunity against asexual lysate). In ( **B**), Kaplan-Meier curves for children with high and low circumsporozoite protein CSP responses and gliding inhibition (GI) phenotypes are presented (N = 51); in ( **C**), curves for participants categorized based on their responses to asexual stage antigens (AL) are shown (N = 51). In both panels ( **B**) and ( **C**), the y-axis corresponds to the percentage of the population uninfected at different time points.

For each immunological assay, ELISA or sporozoite gliding motility assays, children were categorized in two groups: participants with high antibody responses (or high sporozoite gliding inhibition activity) were those with assay values higher than the study population median ( Figure 3A); children considered to have low antibody responses or low sporozoite gliding inhibition capacity had values lower than the median. Based on this categorization, study subjects with high CSP responses and those whose plasma inhibited sporozoite gliding movement acquired blood-stage *P. falciparum* infection (qPCR-based parasitaemia ≥ 0.1 parasites/μl) later compared to children with lower CSP responses and less efficient gliding inhibitory activity ( Figure 3B; P=0.05 and P=0.01 for CSP responses and sporozoite gliding inhibition, respectively). High anti-LSA-1 antibody levels, on the other hand, did not influence time to infection (P=0.31). Blood-stage immunity, i.e. high response in the asexual stage lysate assay, was also associated with longer time to PCR-detected infection ( Figure 3C, P=0.005). We repeated these analyses excluding children with haemoglobin S and haemoglobin C mutations, as these conditions might influence both immunity ^15^ and parasite carriage ^16, 17^. Despite the limited number of individuals included in this analysis (n=36), similar results were obtained (P=0.01 for both CSP and asexual stage lysate assays and P=0.05 for the sporozoite gliding inhibition assay). Additional analyses (Extended data ^14^) show that functional responses against pre-erythrocytic stages were also associated with infection risk when different parasite density thresholds are used to define infection.

Since children with high CSP responses were more likely to have high antibody responses to asexual blood stage parasite lysate compared to low CSP responders (60% versus 38.5%, respectively; P=0.12), multivariate Cox proportional hazards models were fit to assess the mutually adjusted effects of these immune responses on time to infection detection ( Table 2). In a model that also included the results of the asexual stage lysate assay, the relationship between anti-CSP responses and time to malaria infection was not statistically significant. Despite a clear trend for a protective effect, the association between inhibition of *in vitro* sporozoite gliding motility and time to falciparum infection also did not reach statistical significance after adjustment for blood-stage immunity (hazard ratio, 0.55; 95% CI, 0.29–1.01).

**Table 2.** Multivariate Cox proportional hazards models for time-to-infection outcome.

Variable	Hazard ratio	95% CI	P-value
**Model I**			
High CSP response	0.62	0.34–1.15	0.13
High response to asexual stage lysate	0.49	0.27–0.89	0.02
**Model II**			
High gliding inhibition activity	0.55	0.29–1.01	0.06
High response to asexual stage lysate	0.52	0.29–0.94	0.03

CI, confidence interval; CSP, circumsporozoite protein.

## Discussion

In this longitudinal study in Burkina Faso, we analysed the associations between functional immune responses against pre-erythrocytic stages of malaria and the incidence of qPCR-detected infections in children aged 5–10 years. We observed that (i) schoolchildren develop antibody responses that can interfere with sporozoite motility and infectivity *in vitro*, and (ii) those children with antibodies that more efficiently reduced *in vitro* sporozoite gliding motility remained uninfected for longer periods of time. Our epidemiological data together with the *in vitro* data provide evidence that there is partially effective pre-erythrocytic immunity that influences individual level infection incidence, although we cannot exclude the contribution of immunity against asexual blood-stage antigens to this observation.

Sterile protection against malaria can be readily demonstrated in human and animal experimental models ^7, 18, 19^, but not following natural malaria exposure ^13^. Field studies showed conflicting evidence on whether naturally acquired immunity can result in delays to patency and showed no evidence for sterile immunity completely preventing the appearance of parasites in the blood stream of exposed individuals ^11, 13^. We selected children aged 5–10, who have lower blood stage immune responses (a potential confounder when studying pre-erythrocytic immunity) compared to semi-immune adults, and who allow repeated blood sampling compared to toddlers. Since submicroscopic infections are prevalent in the area ^20^, we used a curative dose of antimalarials to clear possible sub-patent infections and, upon confirmation that children were parasite-free at the start of the transmission season, assessed the infection incidence by sensitive molecular assays. Our finding that 98% of the cohort became infected with *P. falciparum* within 3 months confirms the high force of infection in the area.

Consistent with previous sero-epidemiological studies ^21– 24^, our results indicate that antibodies to *P. falciparum* sporozoite and liver-stage antigens are acquired following natural exposure. Antibody titres to the CSP antigen were on average low in our cohort and not significantly different from malaria-naive donors. These low titres could be related to the timing of the study recruitment (prior to the transmission season after ~7 months of very low malaria exposure), which would imply that responses to CSP are short lived. The antibody levels could also have been influenced by repeated blood-stage infections in our cohort, which may have suppressed immune responses against the pre-erythrocytic stages ^25^, although, in theory, responses to both LSA-1 and CSP would be affected. To assess the contribution of pre-erythrocytic humoral immunity to protection, previous studies have related antibodies at baseline with time-to-infection after parasite clearance with antimalarials ^12^. A significant challenge in these studies is that both protective immunity and cumulative exposure increase with age, and so it is often unclear whether measured responses mediate protection or are merely a marker of past exposure ^26, 27^. In an attempt to move beyond indirect epidemiological associations, we explored functional anti-sporozoite immunity by assessing the ability of plasma to inhibit sporozoite gliding motility ^28^ and hepatocyte invasion ^29^ and related these *in vitro* phenotypes to field findings. We observed that CSP IgG antibodies showed a strong positive association with sporozoite gliding motility inhibition. It has been demonstrated that *Plasmodium* parasites use the system of adhesion-based motility, gliding, to actively penetrate host cells ^30^; and that the invasive ability of sporozoites is associated with their motility ^31^. In agreement with these findings, the data presented here suggest that *in vitro* gliding inhibition by naturally acquired antibodies may be a useful surrogate marker for *in vitro* hepatocyte invasion. In an immuno-epidemiological study undertaken in Indonesia, hepatocyte invasion inhibition was associated with higher anti-CSP antibody titres ^32^. In our cohort, subjects with high CSP responses (i.e. higher than the study population median) and those whose plasma more efficiently inhibited sporozoite gliding movement developed *P. falciparum* infection later compared to children with lower responses. To our knowledge, this is the first study to show that there are functional antibodies against pre-erythrocytic malaria stages in malaria-exposed children.

Our study has several limitations. We observed that high responses in the asexual stage lysate assay with unknown functionality were also associated with longer time to infection. We thus cannot rule out a supportive role for asexual antibody responses in the observed associations ^33^. Indeed, as pointed out by one of the reviewers of the first version of this manuscript, 17/29 (58.6%) children with high anti-sporozoite responses in either CSP-specific or sporozoite gliding assays had above-median asexual lysate responses versus 8/22 (36.4%) children with below-median anti-sporozoite activities. We believe it is likely that the functionally important pre-erythrocytic antibody responses that we quantified here are acquired alongside anti-blood-stage antibodies. In addition, a recent study in the same geographical area demonstrated that heterogeneity in mosquito exposure contributes considerably to heterogeneity in parasite inoculation risk ^34^. In the current study, we used delayed time to blood-stage infection as a simplistic indicator of partial protection, which fails to take into account variation in exposure. In an ideal approach, we would have been able to quantify malaria exposure at an individual level, which may involve linking of blood meals in household-caught mosquitoes to household occupants and determining sporozoites in the salivary glands of these mosquitoes ^35^. Measuring exposure at individual level in such an approach will help to shed further light on pre-erythrocytic immunity in naturally exposed individuals. With respect to discriminating between pre-erythrocytic and blood-stage immunity, a valuable but laborious approach would be to examine the observed relationships in a larger cohort that allows stratification based on similar blood stage immunity but different levels of pre-erythrocytic immunity at baseline and that provides sufficient power to detect weaker associations. Another limitation of our study is that we were only able to determine humoral responses. Cellular responses to pre-erythrocytic stages have been implicated in malaria protection in multiple studies ^36, 37^. It is conceivable that by quantifying both antibody and cellular responses we would be able to better define natural immunological phenotypes associated with differential malaria risk ^38^. Immunity to mosquito saliva might have also influenced sporozoite invasion although the magnitude of such an effect compared to the established impact of anti-sporozoite responses is currently unknown and it is currently unknown whether this would require responses to antigens that are conserved between *A. stephensi* mosquitoes used for sporozoite production and vectors that study participants were naturally exposed to 39.

In summary, in our cohort of children, anti-CSP antibodies were strongly associated with *in vitro* sporozoite gliding inhibition and hepatocyte invasion inhibition. Children with functional anti-sporozoite antibody responses had a longer time to *P. falciparum* infection compared to children with lower functional responses, suggesting that these *in vitro* assays are relevant to understand natural protection. The partial protection (i.e., delay in infection) observed in our study does not prevent individuals from becoming infected during an entire transmission season, but reduces infection incidence and consequently needs to be considered in epidemiological studies aiming to understand malaria risk heterogeneity and in malaria vaccines trials. Identifying host or parasite factors linked to these functional immunological phenotypes and characterizing how these phenotypes change with cumulative exposure to malaria parasites will help the understanding of why natural immunity against pre-erythrocytic stages is incomplete.

## Methods

### Study design

This study was performed from June to December 2015 in the village of Balonghin in the Saponé health district, Burkina Faso, which is exposed to intense and seasonal *P. falciparum* transmission ^20^. Written informed consent was provided by the parent or guardian of each child. The study was approved by the ethics committees of the London School of Hygiene and Tropical Medicine (reference number 9008) and the Ministry of Health in Burkina Faso (reference number 2015-3-033). Children aged 5–10 years with haemoglobin levels above 8 g/dl and no *Plasmodium* parasites detected by microscopy were eligible. DHA-PQ was used to clear sub-microscopic infections. At 3 weeks (20–22 days) after treatment, finger-prick blood samples were collected to ensure parasite negativity by nested PCR ^40^ prior to formal enrolment into the cohort. Citrated plasma samples were collected before treatment using citrated vacutainer cell preparation tubes (CPT vacutainers, Becton Dickinson), stored at -80°C and used for malaria-antigen-specific IgG ELISAs and sporozoite assays. Peripheral blood mononuclear cells (PBMC) were also collected, but were lost due to the inability to maintain liquid nitrogen supplies during civil unrest in Ouagadougou. Following enrolment, participants were examined during weekly visits, when finger-prick samples were collected for *P. falciparum* nested PCR that was performed within 48 hours. Following parasite detection, finger-prick blood samples were collected every day for 1 week, and every week afterwards, up to 35 days after parasite detection. Study participants were closely monitored for the development of malaria symptoms. Artemether-lumefantrine was given upon the detection of symptoms or 35 days after initial detection of infection by nested PCR, whichever came first. For the current analyses, only the time to first infection detection was used and related to baseline immunological assays.

### Molecular analyses

Nucleic acids from 100 μl whole-blood samples stored in RNAprotect Cell Reagent were extracted using MagNAPure LC automatic extractor (Total Nucleic Acid Isolation Kit—High Performance, Roche Applied Science) and used for qPCR targeting *18S* rRNA ^41^. Genomic DNA from the same extraction was used to test for human haemoglobinopathies haemoglobin S and C ^42^.

### Parasite culture and generation of P. falciparum-infected mosquitoes

As source of sporozoites, *Anopheles stephensi* mosquitoes were infected by standard membrane feeding on *P. falciparum* NF54 gametocyte cultures ^43^. Salivary glands from infected mosquitoes were dissected, collected in Leibovitz culture medium (Lonza) without serum (supplemented with 1% penicillin-streptomycin and 1% L-glutamine for *in vitro* gliding motility assays), and homogenized in a homemade glass grinder. The number of sporozoites was counted in a Bürker-Türk counting chamber using phase contrast microscopy ^18^.

### Human hepatoma HC-04 cell line

The HC-04 human hepatoma cell line ^29^ was acquired through MR4 as part of the Biodefense and Emerging Infections Research Resources Repository (BEI Resources). Hepatoma cells (referred to as hepatocytes) were cultured in Dulbecco’s Modified Eagle Medium (DMEM)/Ham’s F-12 nutrient mixture medium (GIBCO) supplemented with 10% heat-inactivated fetal bovine serum (FBS, GIBCO), 1% glutamine and 1% penicillin/streptomycin (GIBCO) at 37°C in an atmosphere of 5% CO _2_.

### Enzyme-linked immunosorbent assays and sporozoite opsonization assays

Levels of antibodies were determined to circumsporozoite protein (CSP: full-length *P. falciparum* NF54 CSP with repeats, produced in *E. coli* by Gennova Biopharmaceuticals Ltd., Pune, India), LSA-1 (LSA-NRC construct expressed in *E. coli*) and asexual lysate using previously reported standardized enzyme-linked immunosorbent assays (ELISAs) ^44, 45^ in naturally exposed children (n=51) and malaria-naive European donors (n=9). Antibody levels were calculated in relation to the positive control (hyperimmune plasma pool from Tanzania) that was set at 100 arbitrary units (AU) using Auditable Data Analysis and Management System (ADAMSEL, version 1.1) ^19^.

Recognition of whole sporozoites by naturally acquired IgG and IgM antibodies was determined by an *in vitro* flow-cytometry-based antibody opsonization assay that was presented in detail elsewhere ^46^. Due to limited plasma availability and available sporozoite numbers, 16 naturally exposed children with highest (8) and lowest (8) gliding activity were selected for sporozoite opsonization assays and invasion assays, allowing us to investigate potential correlations. Flow cytometric analysis was performed with a LSRII flow cytometer (BD BioSciences); data analysis by FlowJo software (version 10.0.8, Tree Star).

Malaria-naive donors are healthy malaria-naive European volunteers who participated in CPS-immunization trial (immunization of malaria-naive human volunteers under chloroquine prophylaxis with sporozoites delivered by mosquito bites) at the Radboud University Medical Center (Nijmegen, The Netherlands) ^47^. Written informed consent was obtained from these individuals including for their samples to be stored and used in additional immunological experiments. Pre-immunization samples collected before the CPS-immunization were used for analysis of malaria antigen-specific antibody levels.

### *In vitro* sporozoite gliding motility assay

Prior to *in vitro* sporozoite assays, plasma aliquots were heat-inactivated for 30 minutes at 56°C, centrifuged at 13,000 rpm for 5 minutes at room temperature and kept at 4°C. Flat-bottom optical-bottom 96-well plates with cover glass base were incubated overnight at 4°C with an anti-CSP monoclonal antibody (produced at Radboudumc Nijmegen, Netherlands ^48^) 3SP2; 5 μg/ml in PBS). Following incubation, wells were washed twice with 150 µl/well PBS, blocked for 20 minutes at room temperature with 100 μl/well Leibovitz medium (Lonza) supplemented with 1% penicillin-streptom **y**cin (GIBCO), 1% L-glutamine (GIBCO) and 10% heat-inactivated FBS(GIBCO). *P. falciparum* NF54 sporozoites (100 µl) were pre-incubated with citrated samples (70 µl; 40% final concentration) for 30 minutes at room temperature and added to each well in triplicate (50 μl/well) at a concentration of 10,000 sporozoites/well. Sporozoites were allowed to glide for 90 minutes at 37°C, 98% humidity, 93% N _2_, 4% CO _2_ and 3% O _2_. Wells were washed thrice with 100 μl/well PBS and gliding trails were fixed for 15 minutes at room temperature with 4% paraformaldehyde (Affymetrix). Following fixation, wells were washed thrice with 100 μl/well PBS and blocked with 150 μl/well 10% FBS/PBS for 20 minutes at room temperature. Subsequently, gliding trails were stained for 1 hour at room temperature with 50 µl/well 5 µg/ml biotinylated anti-CSP monoclonal antibody (anti-CSP 3SP2 antibodies were produced at Radboudumc, Nijmegen, the Netherlands ^48^), followed by a wash step (thrice with PBS) and a 1 hour incubation at room temperature with 50µl/well 10 µg/ml streptavidin-Alexa Fluor-594 (Life Technologies) diluted in 10% FBS in PBS. Subsequently, wells were washed thrice with 100 μl/well PBS and stored in 150 μl/well PBS at 4°C in the dark until analysis. Gliding trails were imaged automatically with the BioTek Cytation cell imager (25 images per well at 200x magnification) and images were analysed automatically by FIJI software (under ImageJ version 2.0.0-rc-68/1.52h) with Otsu’s thresholding ^28^. Results were plotted in GraphPad Prism version 5.03.The number of pixels present on a stitched image made from 25 individual pictures taken per well is a measure of the amount of shed CSP in that particular well and therefore, differences in the number of pixels can be interpreted as differences in sporozoite gliding trail surface ^28^.

### *In vitro* sporozoite infectivity assay of a human hepatoma cell line

Neutralization of *P. falciparum* sporozoite hepatocyte invasion by naturally acquired antibodies was assessed in a flow-cytometry-based *in vitro* invasion assay, as previously described with small adaptations ^46^. Briefly, freshly dissected *P. falciparum* NF54 sporozoites were added to heat-inactivated plasma samples (10% final concentration) from malaria-naive or malaria-exposed individuals and pre-incubated for 30 minutes at 4°C. Subsequently, the sporozoite-plasma mixtures (5.10 ^4^ sporozoites in the presence of 10% plasma) were added to HC-04 hepatocytes in 96-well plates. Following 3 hours of incubation at 37°C in 5% CO2, invaded and intracellular sporozoites were stained with an Alexa Fluor 488-conjugated anti-CSP antibody. Flow cytometric analysis was performed with a Gallios (Beckman Coulter) flow cytometer and data were analysed with FlowJo software (version 10.0.8, Tree Star). The percentage of CSP-positive hepatocytes was first corrected for background reactivity by subtracting the background (uninfected HC-04 cells in the presence of 3SP2-Alexa Fluor-488 antibody). The percent invasion inhibition was expressed relative to control IgG.

### Statistical analysis

For analysis of *in vitro* sporozoite data, comparisons between two (controls versus field samples) or multiple groups were performed using Mann-Whitney U-test and Kruskal-Wallis test followed by Dunn’s test between two groups, respectively. The associations between immune responses and malaria infection risk were assessed using survival analysis methods. Log-rank test was used to compare times to infection incidence for individuals with different values of immune phenotypes. Cox survival models were fit to assess the effect of pre-erythrocytic immunity after adjustment for blood-stage immunity; the proportional hazards assumption was tested using Schoenfeld residuals. In these analyses, study participants were considered to have high or low responses (binary explanatory variables) based on the study population median (see Figure S5 in Extended data ^14^). The first scheduled weekly visit or intensive follow-up visit when parasitaemia of at least 0.1 parasites per μl was detected by *18S* qPCR was considered the time of infection incidence. Using this criterion, the median *18S* qPCR-based parasitaemia at infection detection was 2.2 parasites per μl and the interquartile range was 0.4 – 80.4. This threshold of parasitaemia was chosen to minimise false-positive results. In the supplemental material, sensitivity analyses were included that used different cut-offs of *18S* qPCR-based density to determine infection positivity. Stata 14 (StataCorp LP, Texas, USA) and GraphPad Prism software (version 5, GraphPad Software Inc., California, USA) were used for statistical analysis. P<0.05 was considered statistically significant.

## Data availability

### Underlying data

The main dataset relating to the field study contains individual level data and identifying information on participants; as such, this dataset is stored under restricted access and not available through an open-access repository. Requests from researchers to access these data for pooled or meta-analysis should be addressed to the corresponding author ( teun.bousema@radboudumc.nl). However, the dataset used in the survival analysis has been de-identified and is available from the Dryad repository, along with raw ELISA results and sporozoite gliding data. DOI: https://dx.doi.org/10.5061/dryad.n1m33qq ^14^.

Data are available under the terms of the Creative Commons Zero "No rights reserved" data waiver (CC0 1.0 Public domain dedication).

### Extended data

The results of sensitivity analyses (see Results section) and Figure S1– Figure S5 are available from the Dryad repository.

**Figure S1. Correlation analyses of naturally acquired whole sporozoite IgG and IgM antibodies.** (A) Scatter plot of whole sporozoite IgG and IgM antibodies targeting *P. falciparum* NF54 sporozoites is shown (n=17). (B) Correlation analysis for *P. falciparum* NF54 sporozoite and CSP-specific IgG antibodies as determined by ELISAs is shown.

**Figure S2. Antibody specificity and *in vitro* gliding inhibition by naturally acquired antibodies.** (A). Scatter plot of *in vitro* gliding inhibition and CSP-specific IgG antibodies as determined by ELISAs is shown (n=51). Samples selected for additional invasion experiments had either poor (orange) or strong (green) gliding inhibitory activity. Correlation analysis for whole sporozoite (B) IgG or (C) IgM antibodies and *in vitro* gliding inhibition by naturally acquired antibodies was conducted with samples from 16 children. Recognition of *P. falciparum* NF54 sporozoites by whole sporozoite **(D)** IgG and (E) IgM antibodies from naturally exposed children (n= 16) or malaria-naive adults (n=8) was shown as the geometric mean fluorescent intensity (MFI) and divided in subgroups: malaria-naive adults (black), poor (orange) *versus* strong gliding inhibitors (green). Correlation analyses were conducted with Spearman correlation analysis. Comparisons between multiple groups were tested by Kruskal Wallis test.

**Figure S3. Inhibition of *in vitro* sporozoite invasion of hepatocytes by antibodies from children in Burkina Faso**. Gliding motility and invasion of *P. falciparum* NF54 sporozoites pre-treated with plasma from children from Burkina Faso and malaria-naive adults was determined by *in vitro* gliding motility and invasion assays in human hepatoma cells. (A) Scatter plot of *in vitro* gliding and invasion inhibition by naturally acquired antibodies is shown. Additionally, correlation analyses of the percent invasion inhibition with whole sporozoite (B) IgG, (C) IgM antibodies or (D) CSP-specific IgG antibodies is shown. Children whom had poor or strong neutralizing effect on sporozoite infectivity are shown in orange and green circles, respectively.

**Figure S4. CSP antibody levels versus immune responses against asexual stage antigens.** In this figure, the x-axis shows CSP antibody levels; the y-axis represents antibody responses against asexual stage parasites lysate. Both axes are in log-scale; one child with undetectable CSP response is not included in this graph.

**Figure S5. Distribution of immune phenotypes.** The distributions of log _10_-transformed antibody responses (x-axes) against CSP, LSA-1 and asexual blood stage lysate (AL) are presented in panels (A), (B) and (C). The y-axes in these panels represent the percentages of study population with various levels of responses. In (A) and (B), one and seven individuals had undetectable responses and, to be included in this figure, were assigned response values equivalent to half of the lower limit of detection. In (D), the results of gliding assays are presented: the left plot presents the distribution of log _10_-transformed sporozoite gliding surface; the right plot shows gliding inhibition (y-axis) for each study participant (different bars; x-axis). The median gliding inhibition (59.6%) was used to define high and low inhibition in the survival analysis.

DOI: https://doi.org/10.5061/dryad.n1m33qq.2 ^14^.

Data are available under the terms of the Creative Commons Zero "No rights reserved" data waiver (CC0 1.0 Public domain dedication).
