# Supplementary material for: Functional antibodies against Plasmodium falciparum sporozoites are associated with a longer time to qPCR-detected infection among schoolchildren in Burkina Faso
Source: Wellcome Open Res. 2019 May 1;3:159. Originally published 2018 Dec 12. [Version 2] doi: 10.12688/wellcomeopenres.14932.2 (PMC6381444; doi:10.12688/wellcomeopenres.14932.2)
Supplement: Peer review history

# Peer review: Reviewer response for version 2

**DOI:** 10.21956/wellcomeopenres.16607.r35410

**Published:** 2019-05-15

**Authors:** Carlota Dobaño

**Affiliations:** 1 Barcelona Institute for Global Health (ISGlobal), Catalonia, Spain

The authors have addressed the points raised in the review.

I have read this submission. I believe that I have an appropriate level of expertise to confirm that it is of an acceptable scientific standard.

# Peer review: Reviewer response for version 2

**DOI:** 10.21956/wellcomeopenres.16607.r35411

**Published:** 2019-05-13

**Authors:** Silvia Portugal

**Affiliations:** 1 Center for Infectious Diseases, Parasitology, Heidelberg University Hospital, Heidelberg, Germany

The manuscript version following the reviewer's suggestions and comments has improved and I have no problems with the current version.

I have read this submission. I believe that I have an appropriate level of expertise to confirm that it is of an acceptable scientific standard.

# Peer review: Reviewer response for version 1

**DOI:** 10.21956/wellcomeopenres.16280.r34757

**Published:** 2019-02-19

**Authors:** Carlota Dobaño

**Affiliations:** 1 Barcelona Institute for Global Health (ISGlobal), Catalonia, Spain

The study by Barry and colleagues is important to shed more light into the efficiency of naturally-acquired sporozoite immunity to interfere with the parasite development at the pre-erythrocytic stages. Functional antibodies are assessed *in vitro* in plasma from *P. falciparum* exposed children for their capacity to interfere with sporozoite gliding and hepatocyte invasion which are required for progress with their life cycle to liver and blood stages, and these are related to titers. The outcome measured is blood stage parasitemia (delay of appearance of subpatent infections) as no direct measurement of liver parasitemia can be done in humans.

 Some changes are required to clarify some aspects and make the work more scientifically sound:

 ABSTRACT

 In the Abstract background, specify if the statement "it is unclear whether immunity that affects the establishment of infections develops following continuous natural exposure" refers to children or in general, and if it refers to naturally acquired immunity as typically perceived (mostly against blood stages) or here if specific for pre-erythrocytic immunity. This also applies to the main Introduction. Although sterilizing immunity does not develop, parasite densities tend to decrease with age.

 In the Abstract Methods it would be relevant to specify the intensity of malaria transmission and the specific area of study (location) within Burkina Faso, to know how repeatedly are they exposed to Pf. Also clarify if it is qPCR or nested PCR (also in results, it is a bit confusing when each is used). Liver stage antigen: which one? Specify also that the functional sporozoite assays are *in vitro*.

 In the results, mention if CSP antibody titres correlated with hepatocyte invasion and if the latter correlated to delayed time to qPCR +ve.

 INTRODUCTION

 See comments in the Abstract section.

 METHODS

- Can it be clarified if children did not receive antimalarial treatment, if during the weekly monitoring visits the nested PCR (done 48h after bleeding) was Pf positive but they had no symptoms (at least for 35 days)? If so, this was accepted by ethics committees easily? Were the families communicated of a positive PCR reaction in absence of symptoms?
- Indicate where the sporozoites were generated.
- Indicate if and how the patent parasitemias were also monitored and taken into account in the analysis.

RESULTS

- When presenting data on median invasion inhibition, please comment on whether these % are high, moderate or low, and give some references.
- The following statement does not look convincing by looking at the Fig S3A scatter plot despite the P=0.02 and Spearman’s ρ=0.60 that appears driven by exteme values: "There was a positive correlation between gliding and invasion inhibition suggesting that *in vitro* gliding inhibition by naturally acquired antibodies might serve as a good surrogate for *in vitro* hepatocyte invasion inhibition." The correlation in Fig S3B is clearer.
- Fig 3A: interesting to see that there is not always overlap of the three metrics, expected to be discussed. Why was hepatocyte invasion inhibition not included?
- Explain the rationale for categorizing in high and low responders. Was the analysis also done with continuous values but did not yield significant insights?
- The joint analysis of the antibody levels to CSP and blood stage lysate and the functional pre-erythrocytic responses is helpful, but shouldn't there be a model with the 3-4 responses together? If not, explain why.

DISCUSSION

- It is not convincing to say that children 5-10 years in a highly endemic area of Burkina Faso have limited blood stage immunity. They will have quite high antibody responses to blood stage antigens already. Better to reformulate.
- It is surprising the lack of difference in antibody levels for CSP between these children and naive volunteers. Even if CSP is not as highly immunogenic as many blood stage antigens, the responses are usually significant and detectable, even in children. Could there be a problem with the capture antigen? Seasonality by itself may not totally explain this, or it would imply they are very short lived. The potential inhibition by blood stage has been proposed but it would also affect LSA1.
- It is not well discussed how the affection of gliding and invasion before liver stage is detected as delay in positive qPCR in the blood, how do authors think this works

As mentioned, the study is valuable due to the lack of data on functional pre-erythrocytic antibody responses in exposed African children.

I have read this submission. I believe that I have an appropriate level of expertise to confirm that it is of an acceptable scientific standard, however I have significant reservations, as outlined above.

# Response

**Published:** 2019-04-22

**Authors:** Bronner Goncalves

**Affiliations:** London School of Hygiene & Tropical Medicine, UK

***Comment***

***“The study by Barry and colleagues is important to shed more light into the efficiency of naturally-acquired sporozoite immunity to interfere with the parasite development at the pre-erythrocytic stages. Functional antibodies are assessed in vitro in plasma from P. falciparum exposed children for their capacity to interfere with sporozoite gliding and hepatocyte invasion which are required for progress with their life cycle to liver and blood stages, and these are related to titers. The outcome measured is blood stage parasitemia (delay of appearance of subpatent infections) as no direct measurement of liver parasitemia can be done in humans.***

***Some changes are required to clarify some aspects and make the work more scientifically sound:***

***ABSTRACT***

***In the Abstract background, specify if the statement "it is unclear whether immunity that affects the establishment of infections develops following continuous natural exposure" refers to children or in general, and if it refers to naturally acquired immunity as typically perceived (mostly against blood stages) or here if specific for pre-erythrocytic immunity. This also applies to the main Introduction. Although sterilizing immunity does not develop, parasite densities tend to decrease with age.”***

*Answer*

In the sentence mentioned by the reviewer, we refer to the fact that there is little or no evidence that naturally acquired immunity against liver stage parasites blocks initiation of blood-stage infection after parasite inoculation by *Anopheles* mosquitoes, including in adults. The term “establishment of infections” in that sentence is equivalent to “release of merozoites, from hepatocytes, in the blood”. We now modified two similar sentences in the *Abstract* and *Introduction *section:

*“Individuals living in malaria-endemic regions develop naturally acquired immunity against severe malarial disease, but it is unclear whether immunity against pre-erythrocytic stages that blocks initiation of blood-stage infection after parasite inoculation develops following continuous natural exposure.” (Abstract)*

*“Individuals living in malaria-endemic regions can develop naturally acquired immunity against severe malaria disease and death, but it is unclear whether immunity that reduces, entirely or partially, the probability of blood-stage infection after parasite inoculation develops following natural exposure.” (Introduction section)*

***Comment***

***“In the Abstract Methods it would be relevant to specify the intensity of malaria transmission and the specific area of study (location) within Burkina Faso, to know how repeatedly are they exposed to Pf. Also clarify if it is qPCR or nested PCR (also in results, it is a bit confusing when each is used). Liver stage antigen: which one? Specify also that the functional sporozoite assays are in vitro.***

***In the results, mention if CSP antibody titres correlated with hepatocyte invasion and if the latter correlated to delayed time to qPCR +ve.”***

*Answer*

We now mention in the *Abstract* the study area and clarify which liver stage antigen was studied:

*“We cleared schoolchildren living in an area (health district of Saponé, Burkina Faso) with highly endemic seasonal malaria of possible sub-patent infections and examined them weekly for incident infections by nested PCR. Plasma samples collected at enrolment were used to quantify antibodies to the pre-erythrocytic-stage antigens circumsporozoite protein (CSP) and Liver stage antigen 1 (LSA-1). In vitro sporozoite gliding inhibition by naturally acquired antibodies was assessed using Plasmodium falciparum NF54 sporozoites; hepatocyte invasion was assessed using the human HC-04 hepatoma cell line and NF54 sporozoites. The associations between antibody responses, functional pre-erythrocytic humoral immunity phenotypes and time to infection detected by 18S quantitative PCR were studied.”*

We have also included information on the correlation between *in vitro *hepatocyte invasion inhibition and anti-CSP antibody levels:

“ *In vitro hepatocyte invasion was inhibited by naturally acquired antibodies (median invasion inhibition, 19.4% [IQR 15.2-40.9%]), and there were positive correlation s between invasion inhibition and gliding inhibition (P=0.0 05, Spearman’s ρ=0.6 7) and between invasion inhibition and CSP-specific antibodies (P=0.002, Spearman’s ρ=0.76).*”

The hepatocyte invasion inhibition phenotype was not included in the survival analysis as only 16 participants had samples tested with this assay.

***Comment***

***“INTRODUCTION***

***See comments in the Abstract section.”***

*Answer*

We addressed these comments in our answer to the first comment of Reviewer 3.

***Comment***

***“METHODS***

***Can it be clarified if children did not receive antimalarial treatment, if during the weekly monitoring visits the nested PCR (done 48h after bleeding) was Pf positive but they had no symptoms (at least for 35 days)? If so, this was accepted by ethics committees easily? Were the families communicated of a positive PCR reaction in absence of symptoms?”***

*Answer*

In this study, we monitored children with weekly samples to identify new infections during the transmission season. We used nested PCR to detect *early, i.e. low density, infections* and characterize for the first time the evolution of naturally acquired acute infections in partially immune children. Children with parasites detected by nested PCR were closely monitored for development of symptoms and treated with first line therapy when symptoms developed. Burkina Faso’s national malaria guidelines did not include treatment of asymptomatic parasite carriage, consequently initial detection of parasites, often at low levels, by nested PCR was not sufficient to prompt administration of antimalarials. It should be noted, and we have clarified this in the *Methods* section, that children received a long-lasting net at the start of the study and were guaranteed access to care 24 hours per day. Moreover, children were clinically examined on a daily basis in the first week of detection of infection. In this manner, the study adhered to the national guidelines but provided improved (access to) care for all study participants.

Parents and guardians were informed that study participation could involve intensive follow-up if parasites were detected in their child’s blood samples and were oriented to contact the study staff if their child developed symptoms. The study was approved by the ethics committee of the London School of Hygiene and Tropical Medicine (reference number 9008) and the Ministry of Health in Burkina Faso (reference number 2015-3-033). Of note, previous studies with similar design were performed that described the development of malaria symptoms in children after detection of patent infections (e.g. M. A. Missinou, B. Lell, P. G. Kremsner, *Uncommon asymptomatic Plasmodium falciparum infections in Gabonese children*. Clin Infect Dis 2003). In the study by Missinou and colleagues, children were followed every second week for detection of infection by blood smear, and after malaria infection was diagnosed, children were clinically monitored with daily blood smears. In large cohort studies conducted in the context of the International Centers of Excellence for Malaria Research (ICEMR) program in Uganda, participants of all age groups (including much younger and with less prior malaria exposure compared to the current study) are followed every 4 weeks with asymptomatic infections sometimes remaining untreated for more than a year (Moss et al. *Malaria Epidemiology and Control Within the International Centers of Excellence for Malaria Research.* Am J Trop Med Hyg. 2015). An important difference between some of these studies and ours is that we used molecular methods to be able to detect infections from the early, sub-patent, phases and we also characterized malaria infectiousness over time, which will be described in a separate manuscript ( *in preparation*). In conclusion, we appreciate the discussion about the necessity to treat asymptomatic infections and believe this is an important discussion to have, but the current study followed national guidelines and is by no means unique in following asymptomatic infections without treatment but with excellent care.

***Comment***

***“Indicate where the sporozoites were generated.”***

*Answer*

As described in the *Methods* section, *Anopheles stephensi* mosquitoes were infected by standard membrane feeding assays on *P. falciparum* NF54 gametocyte cultures. These experimental mosquito infections were performed in the insectarium of the Department of Medical Microbiology of the Radboud University Nijmegen Medical Centre.

***Comment***

***“Indicate if and how the patent parasitemias were also monitored and taken into account in the analysis.”***

*Answer*

The analysis presented in this manuscript focuses on the influence of immune factors on infection establishment, i.e. appearance of falciparum parasites in the blood. Consequently, all different comparisons presented relate to the first visit of each participant when parasites were detected by sensitive molecular methods. Blood smears were also performed for all study visits, before and after infection detection, but results were not immediately available. The relationship between blood smear results and parasite and gametocyte levels quantified by molecular methods after infection detection will be described in detail in a separate manuscript ( *in preparation*).

***Comment***

***“RESULTS***

***When presenting data on median invasion inhibition, please comment on whether these % are high, moderate or low, and give some references.”***

*Answer*

The assessment of whether *in vitro *hepatocyte invasion inhibition was high, moderate or low requires comparisons of inhibition percentages between different studies, which might be less valid than within-study comparisons between samples from different participants tested with the same protocol. Furthermore, without an established quantitative relationship between the results of this assay and *in vivo *infection incidence, semi-quantitative statements of this type are difficult to interpret. Some studies that performed the same *in vitro *assay were cited:

Behet et al. *The complement system contributes to functional antibody-mediated responses induced by immunization with Plasmodium falciparum malaria sporozoites*. Infect Immun. 2018

Sattabongkot et al. *Establishment of a human hepatocyte line that supports in vitro development of the exo-erythrocytic stages of the malaria parasites Plasmodium falciparum and P. vivax. *Am J Trop Med Hyg. 2006

***Comment***

***“The following statement does not look convincing by looking at the Fig S3A scatter plot despite the P=0.02 and Spearman’s ρ=0.60 that appears driven by extreme values: "There was a positive correlation between gliding and invasion inhibition suggesting that in vitro gliding inhibition by naturally acquired antibodies might serve as a good surrogate for in vitro hepatocyte invasion inhibition." The correlation in Fig S3B is clearer.”***

*Answer*

In our analysis of the correlation between *in vitro *hepatocyte invasion inhibition and *in vitro *sporozoite gliding inhibition, we used the Spearman’s rank correlation method, which assessed the correlation between the ranking of the values in these two phenotypes, rather than the values themselves. As additional descriptive information, we added the following sentence:

“ *7/8 plasma samples from strong gliding inhibitors reduced hepatocyte invasion by 20% or more, whereas only 2/8 poor gliding inhibitors inhibited at least 20% of hepatocyte invasion. There was a positive correlation between gliding and invasion inhibition (Figure S3A in Extended data14, P=0.005, Spearman’s ρ=0.67), suggesting that in vitro gliding inhibition by naturally acquired antibodies might serve as a good surrogate for in vitro hepatocyte invasion inhibition.*”

***Comment***

***“Fig 3A: interesting to see that there is not always overlap of the three metrics, expected to be discussed. Why was hepatocyte invasion inhibition not included?”***

*Answer*

The *in vitro* hepatocyte invasion assay was only performed in a subset of the study population (16 children) and for this reason was not included in this figure and in the survival analysis. The invasion experiments are highly laborious and resource intensive. We used these as supportive experiments in our study, to confirm the relevance of gliding assays, but invasion assays did not form the core of our analysis. We therefore feel that our approach to perform these experiments on a selective number of samples is justified.

***Comment***

***“Explain the rationale for categorizing in high and low responders. Was the analysis also done with continuous values but did not yield significant insights?”***

*Answer*

The survival analysis was only performed with binary variables. The rationale for categorizing the study population in high and low responders, based on the median, was to facilitate interpretation of the results and avoid assumptions about linearity of effect.

***Comment***

***“The joint analysis of the antibody levels to CSP and blood stage lysate and the functional pre-erythrocytic responses is helpful, but shouldn't there be a model with the 3-4 responses together? If not, explain why.”***

*Answer*

Since CSP-specific antibody levels are highly correlated with sporozoite gliding inhibition (see *Results* section), we decided not to include these two variables in the same model. Additionally, the limited sample size also influences the number of predictors that can be reasonably included in our multivariate analyses.

***Comment***

***“DISCUSSION***

***It is not convincing to say that children 5-10 years in a highly endemic area of Burkina Faso have limited blood stage immunity. They will have quite high antibody responses to blood stage antigens already. Better to reformulate.”***

*Answer*

We agree that children in highly endemic areas will have considerable immunity by the age of 5 years. However, our statement compared immunity in children relative to adults: “ *We selected children aged 5–10, who have limited blood stage immunity (a potential confounder when studying pre-erythrocytic immunity) compared to semi-immune adults, …*.”. We have now modified the sentence to avoid misinterpretation:

*“We selected children aged 5–10, who have lower blood stage immune responses (a potential confounder when studying pre-erythrocytic immunity) compared to semi-immune adults, and who allow repeated blood sampling compared to toddlers.”*

***Comment***

***“It is surprising the lack of difference in antibody levels for CSP between these children and naive volunteers. Even if CSP is not as highly immunogenic as many blood stage antigens, the responses are usually significant and detectable, even in children. Could there be a problem with the capture antigen? Seasonality by itself may not totally explain this, or it would imply they are very short lived. The potential inhibition by blood stage has been proposed but it would also affect LSA1.”***

*Answer*

Thank you for these suggestions. The reviewer suggested that suppression of immune responses by blood-stage parasites would, theoretically, affect responses to both antigens, LSA-1 and CSP. We agree and have now modified the *Discussion *section accordingly:

*“These low titres could be related to the timing of the study recruitment (prior to the transmission season after ~7 months of very low malaria exposure) , which would imply that responses to CSP are short lived. The antibody levels could also have been influenced by repeated blood-stage infection s in our cohort, which may have suppressed immune responses against the pre-erythrocytic stages , although, in theory, responses to both LSA-1 and CSP would be affected.”*

The reviewer also suggested that there might have been a problem with the antigen used in the CSP assay. The significant correlations between anti-CSP levels and functional phenotypes (i.e. sporozoite gliding inhibition or hepatocyte invasion inhibition) suggest that this is not the case (or that even if there was an issue, the interpretation of the relative values is still valid), and this is supported by the fact that a subset of children had particularly high antibody responses to CSP (Figure 2A).

***Comment***

***“It is not well discussed how the affection of gliding and invasion before liver stage is detected as delay in positive qPCR in the blood, how do authors think this works”***

*Answer*

We observed an association between sporozoite gliding inhibition and time to parasite positivity by qPCR. This could either reflect an ability of these immune responses to completely neutralize some sporozoite inoculums, leading to a delay in patency because this population would have no merozoites being released into the bloodstream from a first sporozoite inoculum. Alternatively, the number of sporozoites that reach hepatocytes can be reduced by immune responses targeting sporozoites and thus, the number of merozoites released upon completion of the hepatic phase of parasite development. This would result in a lower starting point of blood stage infection and plausibly a longer time to reach detectability of infections by PCR. Lastly, and we believe this is well explained in the revised manuscript, it is possible that the number of released merozoites is unaltered in the population with high sporozoite immunity, but the concurrent higher blood stage immunity dampens parasite growth and results in a longer time to reach detectability of infections by PCR.

# Peer review: Reviewer response for version 1

**DOI:** 10.21956/wellcomeopenres.16280.r34445

**Published:** 2019-02-01

**Authors:** Peter F. Billingsley

**Affiliations:** 1 Sanaria Inc., Rockville, MD, USA

This ms measures antibody responses in Burkinabe children after drug clearance and subsequent *Plasmodium falciparum *infections, then attempts to explain delays in time to infection in terms of antibodies against circumsporozoite protein (CSP) and whole parasites.  The authors demonstrate that, in individuals who show greatest delays in time to parasitemia, there were stronger functional antibody responses against sporozoites which reduced gliding motility and liver cell invasion *in vitro*.  The major question addressed in this ms is whether these anti-sporozoite responses are affecting time to parasitemia in these children.  From the data presented, the answer is “maybe”.  The problem the authors face is that for almost every infection that stimulates anti-sporozoite antibodies, that infection also stimulates blood stage antibodies as the infection progresses.  It is these latter that show the best negative correlation in reduction in time to parasitemia, and while separating out the functional anti-sporozoite effects is informative it is not definitive.  This is illustrated in Figure 3A, where of the 29 participants with anti-sporozoite functional activities in one or both of the assays, 17 also have higher than median anti-blood stage responses.  I do not see a way around this at the participant level and the authors clearly acknowledge this difficulty in their discussion; one is trying to see subtle effects on infection (in this case time to infection no children we uninfected during the study) using markers that autocorrelated and cannot therefore be separated from one another in terms of cause and effect.

 One additional complication is not considered as far as I can tell.  In order to have become infected, these children must have been bitten by mosquitoes.  The mosquito bites induce antibody responses.  The sporozoites used in the assays for gliding motility and hepatocyte invasion were from crude homogenates that contain salivary gland material.  It is possible (though I admit not too likely) that there was some functional interaction between these two.  Could antibodies against ( *Anopheles stephensi*) salivary glands be present in these sera (of children bitten by African anophelines), and if so, could they have affected the *in vitro* functional antibody assays?

 Nevertheless, the ms provides important information concerning anti-sporozoite functional antibodies, even if a clinical outcome (delayed time to parasitemia) cannot be ascribed to them.

**Other points**:

1. Given the comments above and the stronger correlation of blood stage antibodies with delay to parasitemia, the authors should consider changing the abstract (and perhaps the title) to include this information.  It is buried in the paper but should be more explicit.
2. At the start of the introduction, the authors highlight the protective efficacies of RTS,S and PfSPZ Vaccine.  However, they should also note that the outcomes, efficacies are different, RTS,S being time to clinical malaria, PfSPZ Vaccine being time to parasitemia.  This has direct relevance to the ms.
3. Also in the introduction, the authors suggest that there is a discrepancy between protection with sporozoites and naturally induced protection not preventing infection.  However, they do not consider the obvious reason for this – dose.  Mosquitoes deliver a few (median 50 sporozoites per bite) now and again over a transmission season, while the sporozoite vaccines are delivering thousands of sporozoites per dose and defined intervals.
4. The authors say that there are sporozoite-specific IgG and IgM antibodies in the malaria exposed children, but such specificity has not been demonstrated, only reactivity.  The term should be changed.
5. What do the authors mean by the title “Evidence of natural risk-modifying pre-erythrocytic immunity”?
6. The authors note an important variable that could not be accounted for in their studies, namely variability in exposure.  One of the authors has worked on antibody responses to mosquito saliva as a marker for exposure risk: would it not be possible to measure antibody responses against mosquito bites here?

**Minor points**:

- Introduction, paragraph 1. The sentence ending “ but it is unclear whether immunity that prevents or reduces infection incidence develops following natural exposure”. This is a misuse of the term incidence – prevention of infection in one person will reduce incidence in the population.  I think the authors mean “that prevents infection or reduces infection intensity in an individual….”.  In the same paragraph, change the word ‘suggests’ (…suggests that sterilizing immunity…” to demonstrates; I think the evidence is pretty clear).
- Results, paragraph 2.  Should read Parasitological and immunological data from the remaining 51 children followed intensively were…”  What does “data from this child were censored after the onset of symptoms” mean?  Could have any one of many interpretations.
- In the results section “Naturally acquired antibodies in children neutralize in vitro sporozoite gliding motility” the authors should change the term “considerably larger’ for something more scientific (significantly greater”?).
- Authors should give sample sizes (n values) used in the curves for figure 3B,C.
- In the discussion, the authors say “To our knowledge, this is one of the first studies to show that there are functional antibodies against pre-erythrocytic malaria stages in malaria-exposed children.” Shouldn’t the other studies be referenced here?
- The authors should check the axis labeling of figures: for example (% infected) ought to be “Percent children infected with P. falciparum”, in Figure S1 the abbreviations/units are not explained, and so on.

I have read this submission. I believe that I have an appropriate level of expertise to confirm that it is of an acceptable scientific standard.

# Response

**Published:** 2019-04-22

**Authors:** Bronner Goncalves

**Affiliations:** London School of Hygiene & Tropical Medicine, UK

***Comment***

***“This ms measures antibody responses in Burkinabe children after drug clearance and subsequent Plasmodium falciparum infections, then attempts to explain delays in time to infection in terms of antibodies against circumsporozoite protein (CSP) and whole parasites.  The authors demonstrate that, in individuals who show greatest delays in time to parasitemia, there were stronger functional antibody responses against sporozoites which reduced gliding motility and liver cell invasion in vitro.  The major question addressed in this ms is whether these anti-sporozoite responses are affecting time to parasitemia in these children.  From the data presented, the answer is “maybe”.  The problem the authors face is that for almost every infection that stimulates anti-sporozoite antibodies, that infection also stimulates blood stage antibodies as the infection progresses.  It is these latter that show the best negative correlation in reduction in time to parasitemia, and while separating out the functional anti-sporozoite effects is informative it is not definitive.  This is illustrated in Figure 3A, where of the 29 participants with anti-sporozoite functional activities in one or both of the assays, 17 also have higher than median anti-blood stage responses.  I do not see a way around this at the participant level and the authors clearly acknowledge this difficulty in their discussion; one is trying to see subtle effects on infection (in this case time to infection no children we uninfected during the study) using markers that autocorrelated and cannot therefore be separated from one another in terms of cause and effect.***

***One additional complication is not considered as far as I can tell.  In order to have become infected, these children must have been bitten by mosquitoes.  The mosquito bites induce antibody responses.  The sporozoites used in the assays for gliding motility and hepatocyte invasion were from crude homogenates that contain salivary gland material.  It is possible (though I admit not too likely) that there was some functional interaction between these two.  Could antibodies against (Anopheles stephensi) salivary glands be present in these sera (of children bitten by African anophelines), and if so, could they have affected the in vitro functional antibody assays?***

***Nevertheless, the ms provides important information concerning anti-sporozoite functional antibodies, even if a clinical outcome (delayed time to parasitemia) cannot be ascribed to them.”***

*Answer*

Thank you. We agree with this assessment of the difficulty in separating pre-erythrocytic-stage- and blood-stage-specific immune responses. We have now included in the *Discussion* section a statement that emphasizes this point:

*“We observed that high responses in the asexual stage lysate assay with unknown functionality were also associated with longer time to infection. We thus cannot rule out a supportive role for asexual antibody responses in the observed associations. Indeed, as pointed out by one of the reviewers of the first version of this manuscript, 17/29 (58.6%) children with high anti-sporozoite responses in either CSP-specific or sporozoite gliding assays had above-median asexual lysate responses versus 8/22 (36.4%) children with below-median anti-sporozoite activities.”*

The reviewer also suggested that the functional activity of the plasma samples from study participants could be partially related to targeting antigens of salivary gland material rather than targeting sporozoites antigens. This is a very novel idea and an area of research that is both exciting and relatively new. There are indeed some indications that saliva responses may influence both the viability of sporozoites (Dragovic et al. *Immunization with AgTRIO, a Protein in Anopheles Saliva, Contributes to Protection against Plasmodium Infection in Mice.* Cell, Host & Microbe 2018) and (not the topic of this paper) the transmission of parasites from man to mosquito (Yamamoto et al. *Inhibition of Malaria Infection in Transgenic Anopheline Mosquitoes Lacking Salivary Gland Cells*. PLoS Pathogens 2016). How important such responses are compared to parasite-specific responses is unclear. Our assumption, based on the available literature, is that responses against salivary proteins would have a considerably smaller impact on sporozoite invasion compared to anti-sporozoite responses.

Moreover, the *in vitro *work that we have done is based on sporozoites harvested from *Anopheles stephensi*, that is not present in West Africa (see Sinka et al. *A global map of dominant malaria vectors. *Parasites & Vectors. 2012). Whilst many salivary proteins appear to be conserved among *Anophelines*, others are distinct between *Stephensi *and *Gambiae s.l. *(Arca *et al. *BMC Genomics 2017). Responses to conserved salivary proteins would thus have to be functionally important in invasion assays and differentially produced by individuals with high sporozoite responses/low sporozoite responses. Our previous work on the salivary protein gSG6 suggested an early saturation of responses to this protein (Stone *et al. *PLoS ONE 2012) and correlations between asexual, sporozoite and salivary gland responses (Proietti *et al. *Am J Trop Med Hyg 2013), making it difficult to disentangle the different responses. In conclusion, there is no convincing evidence that salivary gland responses could explain our current findings but it is a theoretical possibility that we cannot rule out completely. We have thus added the following underlined sentence to the paragraph where we describe some of the limitations of this study:

*“Another limitation of our study is that we were only able to determine humoral responses. Cellular responses to pre-erythrocytic stages have been implicated in malaria protection in multiple studies. It is conceivable that by quantifying both antibody and cellular responses we would be able to better define natural immunological phenotypes associated with differential malaria risk. Immunity to mosquito saliva might have also influenced sporozoite invasion although the magnitude of such an effect compared to the established impact of anti-sporozoite responses is currently unknown and it is currently unknown whether this would require responses to antigens that are conserved between An. stephensi mosquitoes used for sporozoite production and vectors that study participants were naturally exposed to.”*

***Comment***

***“Other points:***

***Given the comments above and the stronger correlation of blood stage antibodies with delay to parasitemia, the authors should consider changing the abstract (and perhaps the title) to include this information.  It is buried in the paper but should be more explicit.”***

*Answer*

To address this comment and comments from Reviewer 1, we have now modified the *Abstract*:

*“Survival analysis indicated longer time to infection in individuals displaying higher-than-median sporozoite gliding inhibition activity (P=0.01) , although this association became non-significant after adjustment for blood-stage immunity (P = 0.06).*

*“In summary, functional antibodies against the pre-erythrocytic stages of malaria infection are acquired in children who are repeatedly exposed to Plasmodium parasites. This immune response does not prevent them from becoming infected during a malaria transmission season, but might delay the appearance of blood stage parasitaemia. Our approach could not fully separate the effects of pre-erythrocytic-specific and blood-stage-specific antibody-mediated immune responses in vivo; epidemiological studies powered and designed to address this important question should become a research priority.”*

***Comment***

***“At the start of the introduction, the authors highlight the protective efficacies of RTS,S and PfSPZ Vaccine.  However, they should also note that the outcomes, efficacies are different, RTS,S being time to clinical malaria, PfSPZ Vaccine being time to parasitemia.  This has direct relevance to the ms.”***

*Answer*

The efficacy metric used in the PfSPZ vaccine trial is mentioned in the *Introduction* section. We now included information on the outcome used to assess efficacy of the RTS,S vaccine:

*“The most advanced malaria vaccine, RTS,S (trade name, Mosquirix), induces immune responses that target P. falciparum circumsporozoite protein (CSP) , and thereby the pre-erythrocytic stages of malaria , and has been shown to be partially effective in delaying the time to clinical malaria episodes.”*

***Comment***

***“Also in the introduction, the authors suggest that there is a discrepancy between protection with sporozoites and naturally induced protection not preventing infection.  However, they do not consider the obvious reason for this – dose.  Mosquitoes deliver a few (median 50 sporozoites per bite) now and again over a transmission season, while the sporozoite vaccines are delivering thousands of sporozoites per dose and defined intervals.”***

*Answer*

We have now modified the following sentence in the *Introduction* section:

*“The results of this and other vaccine trials and the efficient immunisation of malaria-naive individuals with multiple infected mosquito bites while receiving chloroquine contrast with the limited epidemiological evidence of naturally acquired functional immunity to Plasmodium pre-erythrocytic stages , which could be possibly linked to the lower number of sporozoites in natural parasite inoculations or to the frequency of host-vector contacts. Individuals living in malaria-endemic regions can develop naturally acquired immunity against severe malaria disease and death, but it is unclear whether immunity that reduces, entirely or partially, the probability of blood-stage infection after parasite inoculation develops following natural exposure.”*

***Comment***

***“The authors say that there are sporozoite-specific IgG and IgM antibodies in the malaria exposed children, but such specificity has not been demonstrated, only reactivity. T44he term should be changed.”***

*Answer*

We agree with the reviewer that the specificity of these antibodies has not been demonstrated, and have now modified the title of the sub-section in the manuscript:

“ *Sporozoite-specific IgG and IgM antibodies in malaria-exposed children*”

to:

“ *Naturally acquired IgG and IgM antibodies targeting sporozoites*”

We also modified the following sentence:

*“In addition to antigen-specific assays, IgG and IgM antibodies recognizing Plasmodium sporozoites were quantified using fluorescently labelled anti-IgG and anti-IgM antibodies by flow cytometric analysis. Levels of antibodies targeting whole sporozoite s were significantly higher in the study participants compared to malaria-naive donors ( Figure 2D, E, P=0.01 and 0.04 for IgG and IgM, respectively). Strong correlations between IgG and IgM antibodies targeting whole sporozoites ( Figure S1A in Extended data 14 , P=0.0005, Spearman’s ρ=0.75), and between CSP-IgG antibody levels and whole sporozoite IgG antibodies ( Figure S1B in Extended data 14 , Spearman’s ρ=0.83, P<0.0001) were also observed.”*

We also replaced the expression “ *sporozoite specific*” with more accurate expressions in other paragraphs of the manuscript.

***Comment***

***“What do the authors mean by the title “Evidence of natural risk-modifying pre-erythrocytic immunity”?”***

*Answer*

This sub-section title refers to the observation that functional immune responses against pre-erythrocytic stages were associated with delay in infection appearance. As discussed and addressed in answers to other comments, this evidence is based on the univariate survival analysis. “Risk” here is being used loosely, and not in the epidemiological sense of probability of an event during a specified time period.

***Comment***

***“The authors note an important variable that could not be accounted for in their studies, namely variability in exposure.  One of the authors has worked on antibody responses to mosquito saliva as a marker for exposure risk: would it not be possible to measure antibody responses against mosquito bites here?”***

*Answer*

Although quantification of antibody responses to mosquito saliva antigens would provide some information on exposure to *Anopheles* mosquitoes generally, as shown in a previous study in the same area ( *Guelbéogo et al, Variation in natural exposure to anopheles mosquitoes and its effects on malaria transmission. eLife 2018*), only a small proportion of blood-fed mosquitoes are parasite carriers, and the prevalence of infection in malaria vectors varies from household to household. For these reasons, we believe quantification of immunity against mosquito saliva antigens alone would not adequately adjust for exposure. Moreover, saliva responses are complicated to use in areas of high mosquito exposure. There is considerable evidence for a level of immune tolerance that reduces responses to mosquito saliva proteins in older individuals with high mosquito exposure. This would complicate the interpretation of mosquito salivary gland responses considerably in our cohort experiencing high but heterogeneous mosquito exposure.

***Comment***

***“Minor points:***

***Introduction, paragraph 1. The sentence ending “ but it is unclear whether immunity that prevents or reduces infection incidence develops following natural exposure”. This is a misuse of the term incidence – prevention of infection in one person will reduce incidence in the population.  I think the authors mean “that prevents infection or reduces infection intensity in an individual….”.  In the same paragraph, change the word ‘suggests’ (…suggests that sterilizing immunity…” to demonstrates; I think the evidence is pretty clear).”***

*Answer*

We modified the sentence below for clarification:

*“Individuals living in malaria-endemic regions can develop naturally acquired immunity against severe malaria disease and death, but it is unclear whether immunity that reduces, entirely or partially, the probability of blood-stage infection after parasite inoculation develops following natural exposure.”*

We prefer to keep the word ‘suggests’ in that same paragraph as “high incidence of blood-stage re-infection after effective anti-malarial treatment in adults” is a strong but not conclusive evidence that sterilizing immunity does not develop.

***Comment***

***“Results, paragraph 2.  Should read Parasitological and immunological data from the remaining 51 children followed intensively were…”  What does “data from this child were censored after the onset of symptoms” mean?  Could have any one of many interpretations.”***

*Answer*

In that sentence, we meant that any visits and samples collected after the development of symptoms were disregarded and the child was classified as uninfected during the entire valid follow-up period.

We replaced the first sentence of that paragraph with the following sentence:

*“Parasitological and immunological data from the remaining 51 children followed intensively were analysed.”*

***Comment***

***“In the results section “Naturally acquired antibodies in children neutralize in vitro sporozoite gliding motility” the authors should change the term “considerably larger’ for something more scientific (significantly greater”?).”***

*Answer*

The modified sentence is:

*“To characterize the effect of human plasma on sporozoite gliding motility that is independent of naturally acquired immunity, sporozoites were incubated in the presence of plasma from malaria-naive individuals (n=5) and showed an average gliding trail surface of 1,427(95% CI 646.6-2,207) pixels, significantly greater than the gliding trail surface of sporozoites incubated with 30 μg/ml of a monoclonal anti-CSP antibody, our positive control,141 (95% CI 71.9-225.4) pixels.”*

***Comment***

***“Authors should give sample sizes (n values) used in the curves for figure 3B,C.”***

*Answer*

We have now included this information in the figure legend.

***Comment***

***“In the discussion, the authors say “To our knowledge, this is one of the first studies to show that there are functional antibodies against pre-erythrocytic malaria stages in malaria-exposed children.” Shouldn’t the other studies be referenced here?”***

*Answer*

We modified the sentence:

“To our knowledge, this is the first study to show that there are functional antibodies against pre-erythrocytic malaria stages in malaria-exposed children.”

***Comment***

***“The authors should check the axis labeling of figures: for example (% infected) ought to be “Percent children infected with P. falciparum”, in Figure S1 the abbreviations/units are not explained, and so on.”***

*Answer*

We have now modified the legends, rather than axes’ labels, of the following figures:

Figure 1

*“… The black line represents the percentage of the study population that remained uninfected at different time-points (y-axis).”*

Figure 3

*“…. In (B), Kaplan-Meier curves for children with high and low circumsporozoite protein (CSP) responses and gliding inhibition (GI) phenotypes are presented (N = 51); in (C), curves for participants categorized based on their responses to asexual stage antigens (AL) are shown (N = 51). In both panels (B) and (C), the y-axis corresponds to the percentage of the population uninfected at different time points.”*

# Peer review: Reviewer response for version 1

**DOI:** 10.21956/wellcomeopenres.16280.r34444

**Published:** 2019-01-03

**Authors:** Silvia Portugal

**Affiliations:** 1 Center for Infectious Diseases, Parasitology, Heidelberg University Hospital, Heidelberg, Germany

In this manuscript Barry, Behet and colleagues address an important question aiming to possibly appoint an effective role in protection from clinical malaria to liver-stage immunity acquired naturally in malaria endemic areas. I believe the work presented is a significant contribution to our understanding of naturally acquired immunity to pre-erythrocytic stages of *P. falciparum *and I make a few comments and suggestions that may improve the current version of the manuscript. 

 The data shows that ABs developed during past malaria cases can reduce sporozoite motility and hepatocyte invasion *in vitro *suggesting that ABs acquired during natural infections can reduce new liver-stage infections. However, the contribution of a possible similar effect *in vivo *is, in my view, less clear from the data presented, and more caution may be needed to discuss the results. All the children followed throughout the study became parasite positive by qPCR, indicating that even children with strong inhibiting ABs were unable to block liver-stage infection efficiently. Furthermore, *high response to asexual stage lysate *might be confounding the analysis. I would add to the manuscript (or supplemental) figures the association of CSP ABs with asexual stage lysate ABs, and also the association of whole SPZ ABs with asexual stage lysate ABs, to give the reader an idea of how close these parameters are.

 I also suggest including, if that has not been done already, the *Reported bed net use *in the multivariate analyses, as this could also be a factor increasing time to PCR positive and clinical malaria. 

 The authors cite the study by Tran et al. ^1^ where it was shown that, in Mali, time to PCR positive was independent of age, while time to clinical malaria increased with age, and where as stated *it was concluded that there no or very limited evidence for an age dependent acquisition of immunity protecting from infection. *Similarly, in the present manuscript, *in vitro *functional data of higher humoral response against pre-erythrocytic stages does not (independently of blood-stage immunity) protect from infection. So, I would rephrase the last sentence in first paragraph of the discussion to add a bit more caution in interpreting what may be causing partial protection. 

 At the end of section *Evidence of natural risk-modifying pre-erythrocytic immunity*the authors should, in my view, clearly state that *High gliding inhibition activity*does not independently associate in a statistically significant way, with *protection against falciparum infection*on the multivariate analyses where blood stage immunity was included; the P value is above 0.05 (0.055) and the CI includes 1, making the relative risk not statistically significant.

 I believe the manuscript could be improved by presenting the quantitative analysis of the *18s *qPCR upon first parasite detection and determine if there is a negative association with the inhibitory capacity of the individuals’ ABs. It would also be very interesting to question if time from first PCR positive to time of presentation of symptoms is different between poor and strong *in vitro *inhibitors. If the *in vitro *data showing gliding inhibition and reduced hepatocyte invasion are significant *in vivo*, one would expect a lower inoculum in the liver and thus a lower parasitaemia on the first PCR positive time-point. And then potentially a slower progression to clinical malaria. I believe with the data generated in this manuscript these analyses could be done, and would enrich the story.

 It is not totally clear to me how individuals were selected for the flow cytometry assays. Survival, gliding inhibition, CSP, LSA1 and asexual lysate ELISAs were performed for the 51 participants, but flow cytometry data presented in fig2 D and E was obtained from 17 Burkinabes only; how were those selected and what is their time to PCR+ in the survival analysis. If they are the 8 poor and 8 strong inhibitors as defined by their gliding inhibition it should be stated in the methods (seems to be so, given supFig3, but there is one extra?). 

 I also suggest to pinpoint these 8 poor and 8 strong inhibitors in fig1 so that the reader would be informed of their time to PCR+ and time to malaria symptoms.

 I would be more cautious when citing ref 31, I believe the study by Michael Stewart et al. ^2^ shows that non-motile SPZ are unable to invade, but is not clearly showing a direct association between % of human AB affecting motility and those levels correlating directly with invasion either.

 Minor points:

 In table 1, I would not refer to the 6 children who were PCR positive at the 3 weeks after treatment time-point as *Persisting parasites post-treatment*as I do not think that it can be excluded that the children were re-infected after clearance of PQ.

 I recommend adding a brief description of the method in ref 45 in the section *In vitro sporozoite infectivity assay of a human hepatoma cell line *inmaterial and methods.

 On page 3 below table 1 there is mention to *field PCR *which may be a mistake.

 The data from the *in vitro *gliding inhibition by LSA IgG seems to be not shown. I think it should be clarified in the text that that is indeed the case. Likewise, if the LSA-1-specific IgG antibodies correlation with sporozoite invasion inhibition is data not shown I would clearly state it in the text.

 In figS2A I would specify that is IgG in the figure x axis and use the label CSP IgG titer instead of CSP antibody titer.

 Figure S3D is called before Figure S3C, I would call figures in ascending and alphabetical order instead.

I have read this submission. I believe that I have an appropriate level of expertise to confirm that it is of an acceptable scientific standard, however I have significant reservations, as outlined above.

## References

[1] TranTMLiSDoumboSDoumtabeDHuangCYDiaSBathilyASangalaJKoneYTraoreANiangalyMDaraCKayentaoKOngoibaADoumboOKTraoreBCromptonPD: An intensive longitudinal cohort study of Malian children and adults reveals no evidence of acquired immunity to Plasmodium falciparum infection. *Clin Infect Dis*.2013;57(1) : 10.1093/cid/cit174 40-7 10.1093/cid/cit174 23487390PMC3669526

[2] StewartMJNawrotRJSchulmanSVanderbergJP: Plasmodium berghei sporozoite invasion is blocked in vitro by sporozoite-immobilizing antibodies. *Infect Immun*.1986;51(3) :859-64 351243610.1128/iai.51.3.859-864.1986PMC260977

# Response

**Published:** 2019-04-22

**Authors:** Bronner Goncalves

**Affiliations:** London School of Hygiene & Tropical Medicine, UK

We would like to thank the reviewers for their insightful comments and suggestions. The modifications we made to address their comments improved the quality of our manuscript and clarified the interpretation of our results.

Reviewer 1

***Comment***

***“In this manuscript Barry, Behet and colleagues address an important question aiming to possibly appoint an effective role in protection from clinical malaria to liver-stage immunity acquired naturally in malaria endemic areas. I believe the work presented is a significant contribution to our understanding of naturally acquired immunity to pre-erythrocytic stages of P. falciparum and I make a few comments and suggestions that may improve the current version of the manuscript.***

***The data shows that ABs developed during past malaria cases can reduce sporozoite motility and hepatocyte invasion in vitro suggesting that ABs acquired during natural infections can reduce new liver-stage infections. However, the contribution of a possible similar effect in vivo is, in my view, less clear from the data presented, and more caution may be needed to discuss the results. All the children followed throughout the study became parasite positive by qPCR, indicating that even children with strong inhibiting ABs were unable to block liver-stage infection efficiently. Furthermore, high response to asexual stage lysate might be confounding the analysis. I would add to the manuscript (or supplemental) figures the association of CSP ABs with asexual stage lysate ABs, and also the association of whole SPZ ABs with asexual stage lysate ABs, to give the reader an idea of how close these parameters are.”***

*Answer*

In response to this comment and to a comment from Reviewer 2, we modified the *Abstract* to mention that our study did not fully address the correlation between liver-stage- and blood-stage-specific immunities and that additional studies are necessary.

*“Survival analysis indicated longer time to infection in individuals displaying higher-than-median sporozoite gliding inhibition activity (P=0.01) , although this association became non-significant after adjustment for blood-stage immunity (P = 0.06).”*

*“In summary, functional antibodies against the pre-erythrocytic stages of malaria infection are acquired in children who are repeatedly exposed to Plasmodium parasites. This immune response does not prevent them from becoming infected during a malaria transmission season, but might delay the appearance of blood stage parasitaemia. Our approach could not fully separate the effects of pre-erythrocytic-specific and blood-stage-specific antibody-mediated immune responses in vivo; epidemiological studies powered and designed to address this important question should become a research priority.”*

We have also modified the first paragraph of the *Discussion* section:

*“Our epidemiological data together with the in vitro data provide evidence that there is partially effective pre-erythrocytic immunity that influences individual level infection incidence , although we cannot exclude the contribution of immunity against asexual blood-stage antigens to this observation.”*

Additionally, we agree that the inclusion of a figure showing the correlation between liver-stage immune responses and asexual stage lysate responses would be informative for the readers. We included a scatter plot of CSP-specific immune responses versus asexual stage lysate responses (Figure S4).

**“ *Figure S4.*** *CSP antibody levels versus immune responses against asexual stage antigens. In this figure, the x-axis shows CSP antibody levels; the y-axis represents antibody responses against asexual stage parasites lysate. Both axes are in log-scale; one child with undetectable CSP response is not included in this graph.*”

We also modified the legend of Figure 3 to refer to this supplementary figure:

“ *To assess the effect of immune responses on infection risk, children were classified based on whether the results of their assays were higher or lower than the study population median (see Results): in ( A), vertically aligned cells represent the same participant, and orange cells indicate that assay results are higher than the median (see also Figure S4, that shows a scatter plot of CSP antibody levels and immunity against asexual lysate).*”

***Comment***

***“I also suggest including, if that has not been done already, the Reported bed net use in the multivariate analyses, as this could also be a factor increasing time to PCR positive and clinical malaria.”***

*Answer*

The analysis in this manuscript concerns time to infection detection, regardless of whether symptoms are present or not. In this cohort, reported bed net use at enrollment had no effect on time to infection (hazard ratio 1.16, 95% CI [0.66 – 2.03], P = 0.61). In analyses that included reported bed net use at enrollment and either CSP immune responses or sporozoite gliding inhibition phenotype as binary variables, neither the hazard ratio point estimates nor the confidence intervals changed significantly (data not shown). Of note, only data on reported bed net use were collected and it is evident that this is an imprecise indicator of personal protection.

***Comment***

***“The authors cite the study by Tran et al. where it was shown that, in Mali, time to PCR positive was independent of age, while time to clinical malaria increased with age, and where as stated it was concluded that there no or very limited evidence for an age dependent acquisition of immunity protecting from infection. Similarly, in the present manuscript, in vitro functional data of higher humoral response against pre-erythrocytic stages does not (independently of blood-stage immunity) protect from infection. So, I would rephrase the last sentence in first paragraph of the discussion to add a bit more caution in interpreting what may be causing partial protection.”***

*Answer*

Our multivariate analyses showed that the effects of functional phenotypes became non-significant after adjustment for blood stage immunity. However, we disagree with the reviewer that we can conclude the opposite, i.e. that “higher humoral response against pre-erythrocytic stages does not (independently of blood-stage immunity) protect from infection”. The study was not designed, and might not have been powered, to assess the separate effects of the correlated pre-erythrocytic-stage and blood-stage immunities on malaria infection establishment. A study with a larger sample size, where a sufficient number of individuals would have relatively high liver-stage immunity and relatively low asexual responses, and vice-versa, would be more suitable to address this question.

We have modified the *Discussion* section and the *Abstract* to mention that our approach did not fully separate the effects of these two types of immunity (see response to the first comment).

***Comment***

***At the end of section Evidence of natural risk-modifying pre-erythrocytic immunity the authors should, in my view, clearly state that High gliding inhibition activity does not independently associate in a statistically significant way, with protection against falciparum infection on the multivariate analyses where blood stage immunity was included; the P value is above 0.05 (0.055) and the CI includes 1, making the relative risk not statistically significant.***

*Answer*

With the small sample size, we believe a very strong focus on the P-value threshold of 0.05 is potentially problematic. In general, the P-value and strict thresholds for interpreting significance are criticized by many (e.g. Greenland et al. Eur J Epidemiol 2016; 31: 337-50; Lang *et al. *Epidemiology 1998; 9:7-8), illustrated also by a recent comment in *Nature* (Amrhein V, Greenland S, McShane B. *Scientists rise up against statistical significance.* Nature 2019). With our small sample size, we agree that all results should be interpreted with caution (also related to the limited sample size, but mostly due to the correlations between blood stage and pre-erythrocytic humoral immunity). The sentence in the *Results* section has now been modified to:

*“ In a model that also included the results of the asexual stage lysate assay, the relationship between anti-CSP responses and time to malaria infection was not statistically significant. Despite a clear trend for a protective effect, the association between inhibition of in vitro sporozoite gliding motility and time to falciparum infection also did not reach statistical significance after adjustment for blood-stage immunity (hazard ratio, 0.55; 95% CI, 0.29–1.01).”*

***Comment***

***“I believe the manuscript could be improved by presenting the quantitative analysis of the 18s qPCR upon first parasite detection and determine if there is a negative association with the inhibitory capacity of the individuals’ ABs. It would also be very interesting to question if time from first PCR positive to time of presentation of symptoms is different between poor and strong in vitro inhibitors. If the in vitro data showing gliding inhibition and reduced hepatocyte invasion are significant in vivo, one would expect a lower inoculum in the liver and thus a lower parasitaemia on the first PCR positive time-point. And then potentially a slower progression to clinical malaria. I believe with the data generated in this manuscript these analyses could be done, and would enrich the story.”***

*Answer*

Thank you, we agree that presenting data on the *18S* qPCR-based parasite levels at the time of infection detection would be informative and have now included the sentence below in the *Methods* section:

*“The first scheduled weekly visit or intensive follow-up visit when parasitaemia of at least 0.1 parasites per μl was detected by 18S qPCR was considered the time of infection incidence. Using this criterion, the median 18S qPCR-based parasitaemia at infection detection was 2.2 parasites per μl and the interquartile range was 0.4 – 80.4. This threshold of parasitaemia was chosen to minimise false-positive results.”*

The median *18S* qPCR parasite density during the first parasite-positive visits of children with higher-than-median sporozoite gliding inhibition activity was 1.0 (interquartile range 0.3 – 28.2) parasites per μl; for those children whose plasma inhibited sporozoite gliding with efficiency below the population median, the parasite density at first detection was 4.7 (median; interquartile range 0.5 – 136.2). The interpretation of associations between these parasite levels and immune phenotypes, however, is not straightforward. Immune responses that reduce liver parasite burden and influence the size of the parasite inoculum in the blood would most likely influence the time required for parasitaemia to reach the detection threshold, but it is not obvious that the parasite levels at which we first detect infection have a direct relation with liver stage immunity. For example, even if after a week (the time between consecutive scheduled visits), parasite levels in blood-stage infections starting on the same day would be higher in children with higher liver-to-blood parasite inocula, if “lower liver-to-blood inoculum” infections remain undetectable, they might be detected at higher levels afterwards.

Regarding the evolution of parasite levels during these natural infections, and the eventual development of symptoms, another manuscript, with a more comprehensive analysis of immune responses against blood stage antigens, is being prepared.

***Comment***

***“It is not totally clear to me how individuals were selected for the flow cytometry assays. Survival, gliding inhibition, CSP, LSA1 and asexual lysate ELISAs were performed for the 51 participants, but flow cytometry data presented in fig2 D and E was obtained from 17 Burkinabes only; how were those selected and what is their time to PCR+ in the survival analysis. If they are the 8 poor and 8 strong inhibitors as defined by their gliding inhibition it should be stated in the methods (seems to be so, given supFig3, but there is one extra?).***

***I also suggest to pinpoint these 8 poor and 8 strong inhibitors in fig1 so that the reader would be informed of their time to PCR+ and time to malaria symptoms.”***

*Answer*

We have rectified this. In the original submission 17 individuals were included in the flow assays (the 16 selected and one additional individual with no hepatocyte invasion data). For consistency, we now presented data for the 8 poor and 8 strong gliding inhibitors with available invasion data in all figures where a selection of the total study population of 51 individuals was used. These 16 individuals were selected based on highest gliding inhibition (84-99%) and lowest gliding inhibition (-34 – 20%), as illustrated in supplementary figure 2B. To ensure consistency, we updated Figure 2, Figure S2 and Figure S3. This had no impact on any of the effects, significance levels changed marginally and have been updated. We have now included this information in the *Methods* section:

*“Recognition of whole sporozoites by naturally acquired IgG and IgM antibodies was determined by an in vitro flow-cytometry-based antibody opsonization assay that was presented in detail elsewhere. Due to limited plasma availability and available sporozoite numbers, 16 naturally exposed children with highest (8) and lowest (8) gliding activity were selected for sporozoite opsonization assays and invasion assays, allowing us to investigate potential correlations.”*

The median time to infection detection in the group of children classified as strong inhibitors of sporozoite gliding motility was 41 days (range 5 – 64 [N = 7]; 1/8 child did not have parasites detected); poor gliding inhibitors (N = 8) had a median time to infection of 34 days (range 13 – 48).

***Comment***

***“I would be more cautious when citing ref 31, I believe the study by Michael Stewart et al. shows that non-motile SPZ are unable to invade, but is not clearly showing a direct association between % of human AB affecting motility and those levels correlating directly with invasion either.”***

*Answer*

We cite the study by Stewart et al in the following sentence:

*“It has been demonstrated that Plasmodium parasites use the system of adhesion-based motility, gliding, to actively penetrate host cells ^30^; and that the invasive ability of sporozoites is directly associated with their motility ^31^.”*

This is an accurate statement about that study, where the authors concluded that “sporozoite invasiveness is associated with sporozoite motility”.

***Comment***

***“Minor points:***

***In table 1, I would not refer to the 6 children who were PCR positive at the 3 weeks after treatment time-point as Persisting parasites post-treatment as I do not think that it can be excluded that the children were re-infected after clearance of PQ.”***

*Answer*

We modified Table 1 to ‘Presence of parasites post-treatment’.

***Comment***

***“I recommend adding a brief description of the method in ref 45 in the section In vitro sporozoite infectivity assay of a human hepatoma cell line in material and methods.”***

*Answer*

*“Neutralization of P. falciparum sporozoite hepatocyte invasion by naturally acquired antibodies was assessed in a flow-cytometry–based in vitro invasion assay as previously described with small adaptations. Briefly, freshly dissected P. falciparum NF54 sporozoites were added to heat-inactivated plasma samples (10% final concentration) from malaria-naive or malaria-exposed individuals and pre-incubated for 30 minutes at 4 ˚C. Subsequently, the sporozoite-plasma mixtures (5.10 ^4 ^sporozoites in the presence of 10% plasma) were added to HC-04 hepatocytes in 96-well plates. Following 3 hours of incubation at 37 ˚C in 5% CO _2_, invaded and intracellular sporozoites were stained with an Alexa Fluor 488-conjugated anti-CSP antibody. Flow cytometric analysis….”*

***Comment***

***“On page 3 below table 1 there is mention to field PCR which may be a mistake.”***

*Answer*

One child in the study did not develop symptoms nor had parasites detected by nested PCR performed in Ouagadougou. We have now modified the sentence to:

*“The median time from confirmation of the absence of parasites (i.e. 3 weeks after anti-malarial treatment) to infection detection by nested PCR or onset of symptoms was 28 days; one child who did not have parasites detected by nested PCR was not included in this calculation.”*

***Comment***

***“The data from the in vitro gliding inhibition by LSA IgG seems to be not shown. I think it should be clarified in the text that that is indeed the case. Likewise, if the LSA-1-specific IgG antibodies correlation with sporozoite invasion inhibition is data not shown I would clearly state it in the text.”***

*Answer*

Although no correlation plot is shown for LSA-1 titers and gliding inhibition, the lack of association with gliding inhibition is included in the *Results* section:

*“In vitro gliding inhibition did not correlate with LSA-1 IgG antibody levels (P=0.11, Spearman’s ρ=0.23)…”*

*“ Sporozoite invasion inhibition correlated with IgG targeting whole sporozoites (Figure S3B in Extended data14, P=0.004, Spearman’s ρ=0.67) but not with whole sporozoite IgM antibody levels ( Figure S3C in Extended data 14 , P=0.13, Spearman’s ρ=0.38). There was a correlation of hepatocyte invasion inhibition with CSP-specific IgG antibodies (Figure S3D in Extended data14, P=0.002, Spearman’s ρ=0.76), but not with LSA-1 specific IgG antibodies (P=0.08, Spearman’s ρ=0.48).”*

In response to the next comment and to comments by another reviewer, we have changed the order of the sentences and removed the word ‘specific’ from the section above and replaced it with “targeting whole sporozoites”.

**Comment**

***“In figS2A I would specify that is IgG in the figure x axis and use the label CSP IgG titer instead of CSP antibody titer.***

***Figure S3D is called before Figure S3C, I would call figures in ascending and alphabetical order instead.”***

*Answer*

We have now modified Figure S2 and rephrased the text so Figure S3C is mentioned before Figure S3D (see answer to previous comment).
